# Telomere-to-telomere assembly of a complete human X chromosome

**DOI:** 10.1038/s41586-020-2547-7

**Published:** 2020-07-14

**Authors:** Karen H. Miga, Sergey Koren, Arang Rhie, Mitchell R. Vollger, Ariel Gershman, Andrey Bzikadze, Shelise Brooks, Edmund Howe, David Porubsky, Glennis A. Logsdon, Valerie A. Schneider, Tamara Potapova, Jonathan Wood, William Chow, Joel Armstrong, Jeanne Fredrickson, Evgenia Pak, Kristof Tigyi, Milinn Kremitzki, Christopher Markovic, Valerie Maduro, Amalia Dutra, Gerard G. Bouffard, Alexander M. Chang, Nancy F. Hansen, Amy B. Wilfert, Françoise Thibaud-Nissen, Anthony D. Schmitt, Jon-Matthew Belton, Siddarth Selvaraj, Megan Y. Dennis, Daniela C. Soto, Ruta Sahasrabudhe, Gulhan Kaya, Josh Quick, Nicholas J. Loman, Nadine Holmes, Matthew Loose, Urvashi Surti, Rosa ana Risques, Tina A. Graves Lindsay, Robert Fulton, Ira Hall, Benedict Paten, Kerstin Howe, Winston Timp, Alice Young, James C. Mullikin, Pavel A. Pevzner, Jennifer L. Gerton, Beth A. Sullivan, Evan E. Eichler, Adam M. Phillippy

**Affiliations:** 1 UC Santa Cruz Genomics Institute, University of California Santa Cruz, Santa Cruz, CA USA; 2 Genome Informatics Section, Computational and Statistical Genomics Branch, National Human Genome Research Institute, National Institutes of Health, Bethesda, MD USA; 3 Department of Genome Sciences, University of Washington School of Medicine, Seattle, WA USA; 4 Department of Molecular Biology and Genetics, Department of Biomedical Engineering, Johns Hopkins University, Baltimore, MD USA; 5 Graduate Program in Bioinformatics and Systems Biology, University of California San Diego, San Diego, CA USA; 6 NIH Intramural Sequencing Center, National Human Genome Research Institute, National Institutes of Health, Rockville, MD USA; 7 Stowers Institute for Medical Research, Kansas City, MO USA; 8 National Center for Biotechnology Information, National Library of Medicine, National Institutes of Health, Bethesda, MD USA; 9 Wellcome Sanger Institute, Hinxton, UK; 10 Department of Pathology, University of Washington, Seattle, WA USA; 11 Cytogenetic and Microscopy Core, National Human Genome Research Institute, National Institutes of Health, Bethesda, MD USA; 12 McDonnell Genome Institute at Washington University, St Louis, MO USA; 13 Undiagnosed Diseases Program, National Human Genome Research Institute, National Institutes of Health, Bethesda, MD USA; 14 Comparative Genomics Analysis Unit, Cancer Genetics and Comparative Genomics Branch, National Human Genome Research Institute, National Institutes of Health, Bethesda, MD USA; 15 Arima Genomics, San Diego, CA USA; 16 Department of Biochemistry and Molecular Medicine, Genome Center, MIND Institute, University of California Davis, Davis, CA USA; 17 DNA Technologies Core, Genome Center, University of California Davis, Davis, CA USA; 18 Institute of Microbiology and Infection, University of Birmingham, Birmingham, UK; 19 DeepSeq, School of Life Sciences, University of Nottingham, Nottingham, UK; 20 Department of Pathology, University of Pittsburgh, Pittsburgh, PA USA; 21 Department of Computer Science and Engineering, University of California San Diego, San Diego, CA USA; 22 Department of Molecular Genetics and Microbiology, Division of Human Genetics, Duke University Medical Center, Durham, NC USA; 23 Howard Hughes Medical Institute, University of Washington, Seattle, WA USA

**Keywords:** Genome informatics, Genome assembly algorithms, DNA sequencing

## Abstract

After two decades of improvements, the current human reference genome (GRCh38) is the most accurate and complete vertebrate genome ever produced. However, no single chromosome has been finished end to end, and hundreds of unresolved gaps persist^1,2^. Here we present a human genome assembly that surpasses the continuity of GRCh38^2^, along with a gapless, telomere-to-telomere assembly of a human chromosome. This was enabled by high-coverage, ultra-long-read nanopore sequencing of the complete hydatidiform mole CHM13 genome, combined with complementary technologies for quality improvement and validation. Focusing our efforts on the human X chromosome^3^, we reconstructed the centromeric satellite DNA array (approximately 3.1 Mb) and closed the 29 remaining gaps in the current reference, including new sequences from the human pseudoautosomal regions and from cancer-testis ampliconic gene families (CT-X and GAGE). These sequences will be integrated into future human reference genome releases. In addition, the complete chromosome X, combined with the ultra-long nanopore data, allowed us to map methylation patterns across complex tandem repeats and satellite arrays. Our results demonstrate that finishing the entire human genome is now within reach, and the data presented here will facilitate ongoing efforts to complete the other human chromosomes.

## Abstract (web summary)

High-coverage, ultra-long-read nanopore sequencing is used to create a new human genome assembly that improves on the coverage and accuracy of the current reference (GRCh38) and includes the gap-free, telomere-to-telomere sequence of the X chromosome.

## Main

Complete, telomere-to-telomere reference genome assemblies are necessary to ensure that all genomic variants are discovered and studied. At present, unresolved areas of the human genome are defined by multi-megabase satellite arrays in the pericentromeric regions and the ribosomal DNA arrays on acrocentric short arms, as well as regions enriched in segmental duplications that are greater than hundreds of kilobases in length and that exhibit sequence identity of more than 98% between paralogues. Owing to their absence from the reference, these repeat-rich sequences are often excluded from genetics and genomics studies, which limits the scope of association and functional analyses^4,5^. Unresolved repeat sequences also result in unintended consequences; for example, paralogous sequence variants incorrectly being called as allelic variants^6^, and the contamination of bacterial gene databases^7^. Completion of the entire human genome is expected to contribute to our understanding of chromosome function^8^, human disease^9^ and genomic variation, which will improve technologies in biomedicine that use short-read mapping to a reference genome (for example, RNA sequencing (RNA-seq)^10^, chromatin immunoprecipitation followed by sequencing (ChIP–seq)^11^ and assay for transposase-accessible chromatin using sequencing (ATAC–seq)^12^).

The fundamental challenge of reconstructing a genome from many comparatively short sequencing reads—a process known as genome assembly—is distinguishing the repeated sequences from one another^13^. Resolving such repeats relies on sequencing reads that are long enough to span the entire repeat or accurate enough to distinguish each repeat copy on the basis of unique variants^14^. The difficulty of the assembly problem and limits of past technologies are highlighted by the fact that the human genome remains unfinished 20 years after its initial release in 2001^15^. The first human reference genome released by the US National Center for Biotechnology Information (NCBI Build 28) was highly fragmented, with half of the genome contained in continuous sequences (contigs) of 500 kb or more (NG50). Efforts to finish the genome^16^, together with the stewardship of the Genome Reference Consortium (GRC)^2^, greatly increased the continuity of the reference to an NG50 contig length of 56 Mb in the most recent release—GRCh38—but the most repetitive regions of the genome remain unsolved and no chromosome is completely represented telomere to telomere. A de novo assembly of ultra-long (greater than 100 kb) nanopore reads showed promising assembly continuity in the most difficult regions^1^, but this proof-of-concept project sequenced the genome to only 5× depth of coverage and failed to assemble the largest human genomic repeats. Previous modelling on the basis of the size and distribution of large repeats in the human genome predicted that an assembly of 30× ultra-long reads would approach the continuity of the human reference^1^. Therefore, we hypothesized that high-coverage ultra-long-read nanopore sequencing would enable the first complete assembly of human chromosomes.

**Extended Data Fig. 1:**
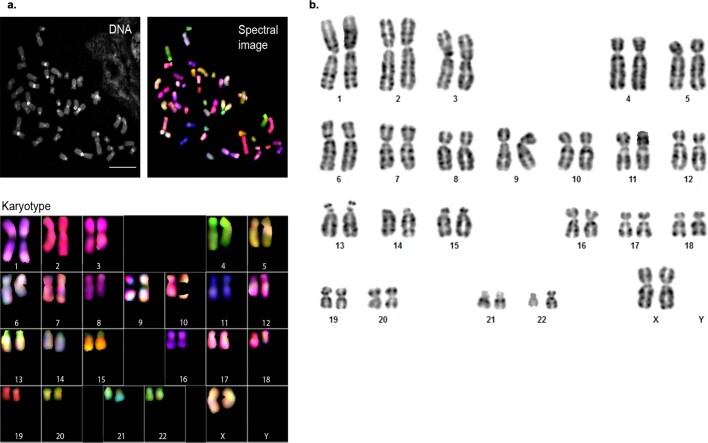
Spectral karyotyping analysis of CHM13 confirmed a normal 46,XX karyotype. **a**, Chromosomes and karyotype of CHM13 cell line at passage 10. Mitotic metaphase spreads were prepared from cells treated with colcemid and processed as detailed in Methods. Spectral karyotyping analysis demonstrated normal. 46,XX karyotype. Representative karyotype is shown from one of ten spreads analysed, all ten reported had similar results. Scale bar, 10 μm. **b**, CHM13 G-banding karyotype. A total of 20 CHM13 metaphase spreads were independently characterized and all showed a similar normal 46, XX female karyotype, as shown.

**Extended Data Fig. 2:**
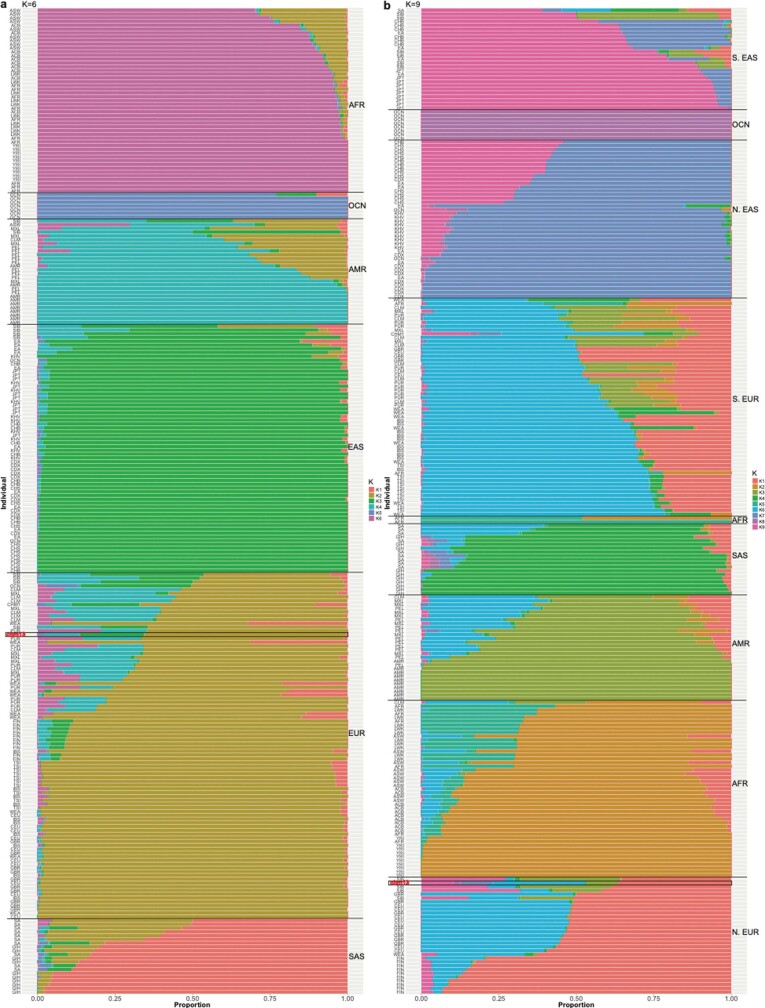
Inferred ancestry of CHM13. **a**, **b**, Proportion of ancestry explained by each cluster as estimated by ADMIXTURE using *K* = 6 (**a**) or *K* = 9 (**b**) for 10 randomly sampled individuals from each population and CHM13. Analysis based on 1,964 unrelated individuals from the 1KG and SGDP. CHM13 is highlighted in red font along with a black bounding rectangle.

To circumvent the complexity of assembling both haplotypes of a diploid genome, we selected the effectively haploid CHM13hTERT cell line for sequencing (hereafter, CHM13)^17^. This cell line was derived from a complete hydatidiform mole (CHM) with a 46,XX karyotype. The genomes of such uterine moles originate from a single sperm that has undergone post-meiotic chromosomal duplication; these genomes are, therefore, uniformly homozygous for one set of alleles. CHM13 has previously been used to patch gaps in the human reference^2^, benchmark genome assemblers and diploid variant callers^18^, and investigate human segmental duplications^19^. Karyotyping of the CHM13 line confirmed a stable 46,XX karyotype, with no observable chromosomal anomalies (Extended Data Fig. 1, Supplementary Note 1). Maximum likelihood admixture analysis^20^ confidently assigns the majority of haplotypes to a European origin, with the potential of some Asian or Amerindian admixture (Extended Data Fig. 2, Supplementary Note 2).

## Highly continuous whole-genome assembly

**Fig. 1:**
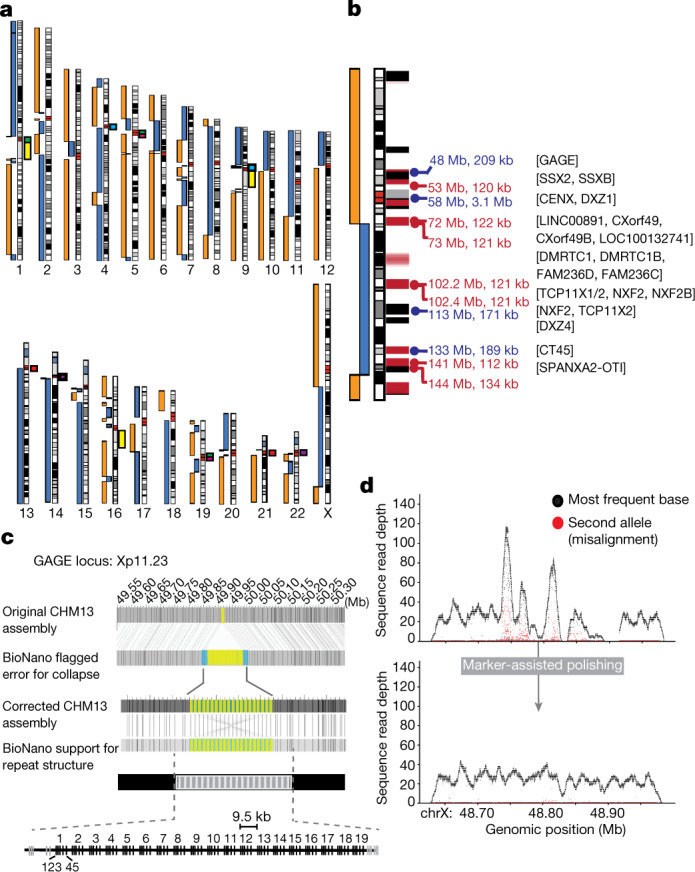
CHM13 whole-genome assembly and validation. **a**, Gapless contigs are illustrated as blue and orange bars next to the chromosome ideograms (highlighting contig breaks). Several chromosomes are broken only in centromeric regions. Large gaps between contigs (for example, middle of chr1) indicate sites of large heterochromatic blocks (arrays of human satellite 2 and 3 in yellow) or ribosomal DNA arrays with no GRCh38 sequence. Centromeric satellite arrays that are expected to be similar in sequence between non-homologous chromosomes are indicated: chr1, chr5 and chr19 (green); chr4 and chr9 (light blue); chr5 and chr19 (pink); chr13 and chr21 (red); and chr14 and chr22 (purple). **b**, The X chromosome was selected for manual assembly, and was initially broken at three locations: the centromere (artificially collapsed in the assembly), a large segmental duplication (DMRTC1B, 120 kb), and a second segmental duplication with a paralogue on chromosome 2 (134 kb). Gaps in the GRCh38 reference (black) and known segmental duplications (red; paralogous to Y, pink) are annotated. Repeats larger than 100 kb are named with the expected size (kb) (blue, tandem repeats; red, segmental duplications). **c**, Misassembly of the GAGE locus identified by the optical map (top), and corrected version (bottom) showing the final assembly of 19 (9.5 kb) full-length repeat units and two partial repeats. **d**, Quality of the GAGE locus before and after polishing using unique (single-copy) markers to place long reads. Dots indicate coverage depth (number of mapped sequencing reads overlapping each base) of the primary (black) and secondary (red) alleles recovered from mapped PacBio HiFi reads (Supplementary Note 4). Because the CHM13 genome is effectively haploid, regions of low coverage or increased secondary allele frequency indicate low-quality regions or potential repeat collapses. Marker-assisted polishing markedly improved allele uniformity across the entire GAGE locus.

High-molecular-weight DNA from CHM13 cells was extracted and prepared for nanopore sequencing using a previously described ultra-long-read protocol^1^. In total, we sequenced 98 MinION flow cells for a total of 155 Gb (50× coverage, 1.6 Gb per flow cell, Supplementary Note 3). Half of all sequenced bases were contained in reads of 70 kb or longer (78 Gb, 25× genome coverage) and the longest validated read was 1.04 Mb. Once we had collected sufficient sequencing coverage for de novo assembly, we combined 39× coverage of the ultra-long reads with 70× coverage of previously generated PacBio data and assembled the CHM13 genome using Canu^21^. Canu selected the longest 30×-coverage ultra-long and 7×-coverage PacBio reads for correction and assembly. This initial assembly totalled 2.90 Gb, with half of the genome contained in continuous sequences (contigs) of length 75 Mb or greater (NG50), which exceeds the continuity of the GRCh38 reference genome (75 versus 56 Mb for NG50). The assembly was then iteratively polished by a series of sequencing technologies in order of longest to shortest read lengths: Nanopore, PacBio and linked-read Illumina. Consensus accuracy improved from 99.46% for the initial assembly to 99.67% after Nanopore polishing and 99.99% after PacBio polishing. Illumina data were used only to correct small insertion and deletion errors in uniquely mappable regions of the genome, which had a marginal effect on the average accuracy but reduced the number of frameshifted genes. Putative misassemblies were identified through analysis of the Illumina linked-read barcodes (10X Genomics) and optical mapping (Bionano Genomics) data not used in the initial assembly. The initial contigs were broken at regions of low mapping coverage and the corrected contigs were then ordered and oriented relative to one another using the optical map. Over 90% of six chromosomes are represented in two contigs and ten are represented by two scaffolds (Fig. 1a).

**Extended Data Fig. 3:**
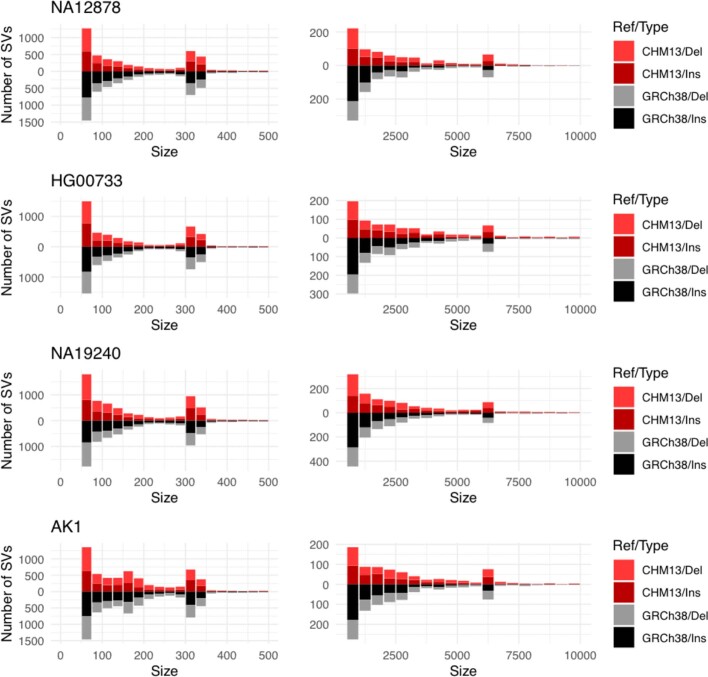
Results of using CHM13 as a reference when describing structural variation. Assemblytics large insertion and deletion calls for four long-read assemblies with respect to CHM13 (in dark red or red) and GRCh38 (in black or grey). Using CHM13 as a reference yields balanced counts of insertions and deletions, whereas an excess of insertion calls is observed when using GRCh38, suggesting a probable deletion bias in GRCh38. SVs, structural variants.

**Extended Data Fig. 4:**
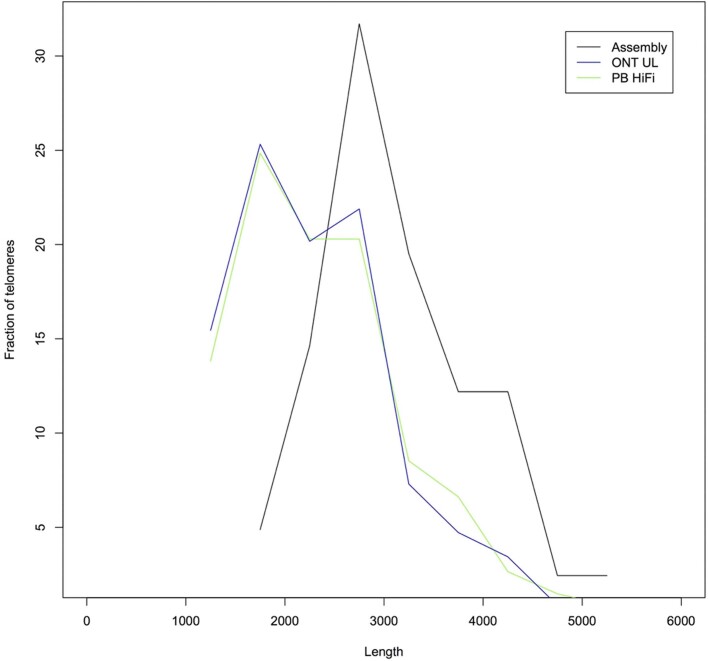
Telomere length in the reads and the assembly. The assembly telomere sizes are consistent with the larger sizes observed in the reads. The shorter peak in telomere length within the reads is probably an artefact of premature read end not the true telomere end. ONT, Oxford Nanopore Technologies; PB, Pacific Biosciences.

The final assembly consists of 2.94 Gb in 448 contigs with a contig NG50 of 70 Mb. A total of 98 scaffolds (173 contigs) were unambiguously assigned to a reference chromosome, representing 98% of the assembled bases. We estimated the median consensus accuracy of this whole-genome assembly to be at least 99.99%, on the basis of both previously finished BAC sequences^22^ and mapped Illumina linked reads (Supplementary Note 4). Although similar to the GRCh38 ungapped length (2.95 Gb), our assembly size is shorter than the estimated human genome size of 3.2 Gb. We estimate approximately 170 Mb of collapsed bases using the Segment Duplication Assembler (SDA) method^19^. Compared to other recent assemblies, we resolved a greater fraction of the 341 CHM13 bacterial artificial chromosome (BAC) sequences that have previously been isolated and finished from segmentally duplicated and other difficult-to-assemble regions of the genome^19^ (Table 1, Supplementary Note 4). Comparative annotation of our whole-genome assembly also shows a higher agreement of mapped transcripts than previous assemblies and only a slightly increased rate of potential frameshifts compared to GRCh38^23^. Of the 19,618 protein-coding genes annotated in the CHM13 de novo assembly, just 170 (0.86%) contain a predicted frameshift, or, if measured by transcripts, only 334 of 83,332 transcripts (0.40%) contain a predicted frameshift (Supplementary Table 1). When used as a reference sequence for calling structural variants in other genomes, CHM13 reports an even balance of insertion and deletion calls (Extended Data Fig. 3, Supplementary Note 5), as expected, whereas GRCh38 demonstrates a deletion bias, as previously reported^24^. Compared to other long-read assemblies, GRCh38 calls twice as many inversions as CHM13 (mean 26 versus 13 inversions per genome), suggesting that some misoriented sequences remain in the current human reference (Supplementary Note 5). Of these inversions, 19 are specific to GRCh38 and not found in 5 recently assembled long-read human genomes (Supplementary Table 5). We identified telomeric sequences within the assembly and the reads (Extended Data Fig. 4, Supplementary Note 4), which were highly concordant in telomere size, and our assembly includes 41 of 46 expected telomeres at contig ends. Thus, in terms of continuity, completeness and correctness, our CHM13 assembly exceeds all previous human de novo assemblies—including the current human reference genome, by some quality metrics (Supplementary Table 2).

**Table 1:** Assembly statistics for CHM13 and the human reference sorted by continuity

Primary technology	Assembly	Size (Gb)	No. of contigs	NG50 (Mb)	BACs resolved (%)	BACs %idy all	BACs %idy uni
56× Illumina linked reads	Supernova (this paper)	2.95	42,828	0.21	17.3	99.975	99.985
76× PacBio CLR	FALCON (ref. ^50^)	2.88	1,916	28.2	36.37	99.981	99.995
24× PacBio HiFi	Canu (ref. ^22^)	3.03	5,206	29.1	45.46	99.979	99.997
Sanger BACs	GRCh38p13 (ref. ^2^)	3.27	1,590	56.4	85.63	99.731^a^	99.768^a^
39× Nanopore ultra-long	Canu (this paper)	2.94	448	70.1	82.11	99.980	99.994

^a^GRCh38 is expected to have a lower identity to BACs derived from CHM13 as it represents a different human genome.

Primary Technology: sequencing technology used for contig assembly. The PacBio CLR assembly was additionally polished using Illumina linked reads. The Nanopore ultra-long assembly was polished with the PacBio CLR and Illumina linked reads. GRCh38 is primarily based on Sanger-sequenced BACs, but has been continually curated and patched since the completion of the human genome project. Assembly: assembler used and reference to the published assembly. Size: sum of bases in the assembly in Gb including N-bases. GRCh38 assembly size includes 110 Mb of alternative (ALT) sequences. No. of contigs: total number of contigs in the assembly; scaffolds were split at three consecutive N-bases to obtain contigs. NG50: half of the 3.09-Gb human genome size contained in contigs of this length or greater in Mb. Supernova NG50 statistics were identical between the two reported pseudo-haplotypes. BACs resolved (%): percentage of 341 ‘challenging’ CHM13 BACs found intact in the assembly. BACs unresolved by the best CHM13 assembly either map across multiple contigs or map to a single contig with large structural variation, indicating an error in either the BAC or whole-genome assembly. BACs %idy all: median alignment accuracy versus all validation BACs. BACs %idy uni: median alignment accuracy versus the 31 validation BACs that occur outside of segmental duplications (Supplementary Note 4).

## A finished human X chromosome

Using this whole-genome assembly as a basis, we selected the X chromosome for manual finishing and validation, owing to its high continuity in the initial assembly; distinctive and well-characterized centromeric alpha satellite array^3,8,25^; unique behaviour during development^26^; and disproportionate involvement in Mendelian disease^3^. The de novo assembly of the X chromosome was broken in three places: at the centromere and at two near-identical segmental duplications of greater than 100 kb (Fig. 1b). The two segmental duplications breaking the assembly were manually resolved by identifying ultra-long reads that completely spanned the repeats and were uniquely anchored on either side, thus allowing for a confident placement in the assembly. Improvements of assembly quality for these difficult regions were evaluated by mapping an orthogonal set of PacBio high-fidelity (HiFi) long reads generated from CHM13^22^ and assessing read depth over informative single-nucleotide-variant differences (Methods). In addition, experimental validation using droplet digital PCR (ddPCR) confirmed that the now-complete assembly correctly represents the tandem repeats of the CHM13 genome, including seven CT47 genes (7.02 ± 0.34 (mean ± s.d.)), six CT45 genes (6.11 ± 0.38), 19 complete and two partial GAGE genes (19.9 ± 0.745), 55 DXZ4 repeats (55.4 ± 2.09) and a 3.1-Mb centromeric DXZ1 array (1,408 ± 40.69 2,057-bp repeats) (Supplementary Note 6).

**Fig. 2:**
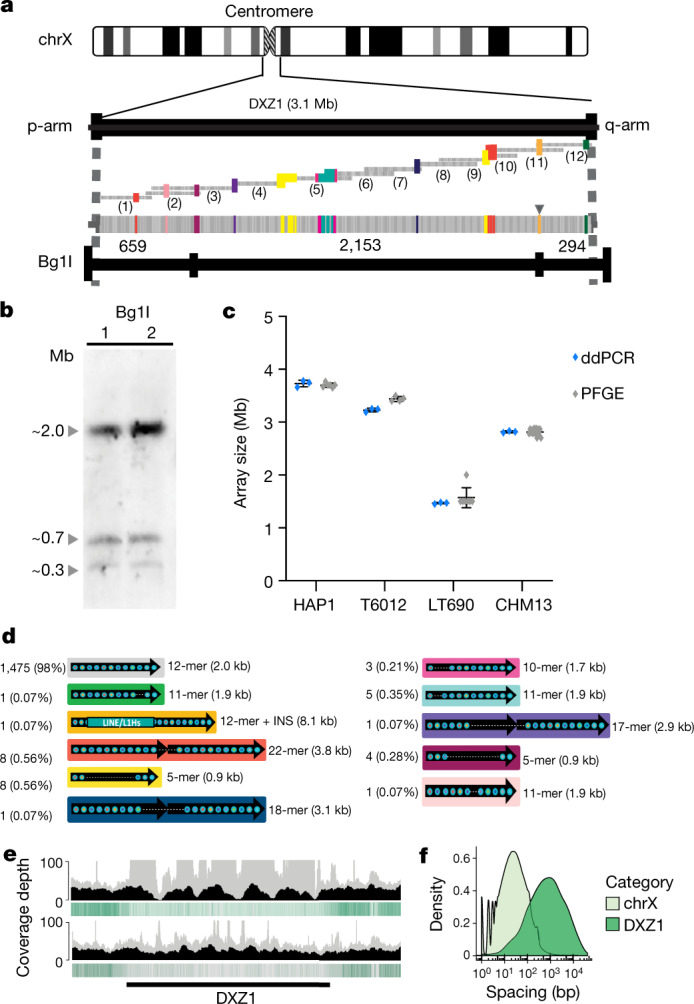
Validated structure of the 3.1-MB CHM13 X-centromere array. **a**, Top, the array, with approximately 2-kb repeat units labelled by vertical bands (grey is the canonical unit; coloured are structural variants). A single LINE/L1Hs insertion in the array is marked by an arrowhead. Bottom, a predicted restriction map for enzyme BglI, with dashed lines indicating regions outside of the DXZ1 array. A minimum tiling path was reconstructed for illustration purposes and was not the mechanism for initial assembly (Extended Data Fig. 5b). **b**, Experimental PFGE Southern blotting for a BglI digest in duplicate (band sizing indicated by triangles; BglI, 2.87 Mb ± 0.16), that matches the in silico predicted band patterns (**a**) for the CHM13 array (experimentally repeated six times with similar results). **c**, Array size estimates were provided using ddPCR (performed in triplicate; mean ± s.d.) optimized against PFGE Southern blots (HAP1, *n* = 6; T6012, *n* = 4; LT690, *n* = 7; CHM13, *n* = 13). **d**, Catalogue of 33 DXZ1 structural variants identified relative to the 2,057-bp canonical repeat unit (grey), along with the number of instances observed, frequency in the array, number of alpha satellite monomers and size. INS, insertion (that is, the 8.1-kb inserted LINE/L1Hs). **e**, Coverage depth of mapped (grey) and uniquely anchored (black) nanopore reads to the DXZ1 array. Marker-assisted polishing (bottom) improves coverage uniformity versus the unpolished (top) assembly. Single-copy, unique markers are shown as vertical green bands, with a decreased but non-zero density across the array. **f**, Distributions show the spacing between adjacent unique markers on chromosome X and DXZ1. On average, unique markers are found every 66 bases on chromosome X, but only every 2.3 kb in DXZ1, with the longest gap between any two adjacent markers being 42 kb.

**Extended Data Fig. 5:**
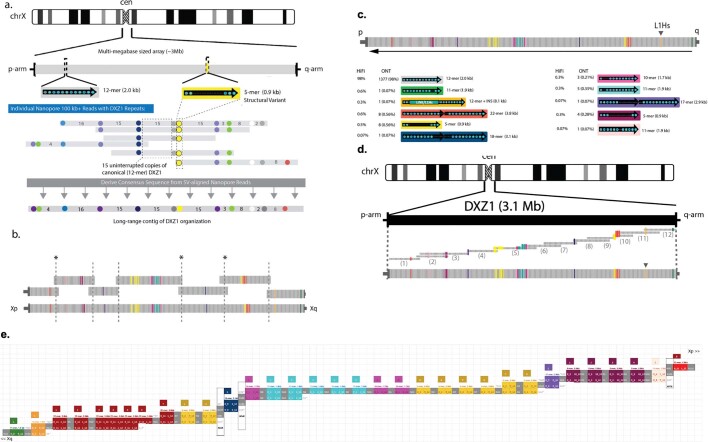
Evaluation of the structure of the X-centromeric satellite array (DXZ1) assembly. **a**, The satellite array on the X chromosome (DXZ1) is defined at the sequence level as a multi-megabase size array of alpha satellite DNA. The canonical repeat of the DXZ1 array is defined by 12 divergent monomers that are ordered to form a larger approximately 2-kb repeating unit, known as a ‘higher-order repeat’ (HOR) (shown in grey, with HOR in black and circles representing each of the twelve approximately 171-bp monomers). The HORs are tandemly arranged into a large, multi-megabase sized satellite array (with previous published PFGE-Southern estimates suggesting a mean of 3 Mb) with a limited number of rearrangements in the HOR repeat structure (as indicated in yellow for a deletion to a 5-mer variant) and nucleotide differences between repeat copies. Our assembly strategy initially identified and annotated all uninterrupted head-to-tail tandem arrays of ‘canonical’ repeats and sites of structural variants in each nanopore read in our DXZ1 library (Methods). The spacing of canonical repeats to flanking structural variants informed the precise alignment between reads. Contigs were generated by taking the consensus of these uniquely placed ultra-long reads. **b**, The T2T-X CHM13 array was originally segmented into seven structural-variant-determined contigs. Ordering and overlap between the contigs was made using shared positions of Duplex-seq DXZ1 kmers and low-coverage (that is, 1–2 reads) support of ultra-long data that confidently spanned contig ordering. Three regions (marked with an asterisk) were only determined by single-nucleotide-variant overlap. We improved the prediction of these overlaps in implementing an orthogonal method, centroFlye, which studies single variant positions in the DXZ1 nanopore reads to guide the final positioning of the overlap between the contigs (and confirm the existing overlap in the region closest to p-arm). **c**, Comparisons with DXZ1 higher-order repeat variant frequency in the nanopore ultra-long-read data HiFi long-read PacBio data were highly concordant. DXZ1 repeat unit variants were predicted in the HiFi dataset using Alpha-CENTAURI^61^. The DXZ1 repeat units, shown as arrows, are composed of 12 smaller approximately 171-bp repeats (indicated as small circles within the arrow). In total, we identified 7,316 DXZ1-containing HiFi reads. We characterized a database of 38,184 (98.2%) full-length DXZ1 canonical 12-mer repeats and 691 HORs with variant repeat structure (1.8%). Changes from the canonical repeat unit are indicated with a dashed line and each structural variant marks a colour, and its positioning within the array assembly is indicated (ordered p-arm to qHiFi-arm) above. The majority of reads were determined to contain purely DXZ1-alpha satellite (7,305/7,316, or 99.85%). Of the remaining reads, ten reads provided evidence for a transition from DXZ1 into the single L1Hs insertion in our assembly. We identified only a single read that we could not assign to our assembly owing to a 902-bp homopolymer ([G]n), which may present a sequencing artefact. **d**, A minimum tiling path was reconstructed for illustration purposes (as shown in Fig. 2a) and was not the mechanism for initial assembly. **e**, DXZ1 read overlap assembly using structural variant overlap and positioning. Read IDs and length are provided from Xp to Xq: (1) ab9c12a7-08db-4524-8332-373129eaa4fb, 442,119 bp. (2) 063fca09-81fc-4c2d-81ad-16fb2bfee76f, 364,710 bp. (3) 3d0fa869-028f-45be-be41-b2487897bb25, 380,361 bp. (4) a5cf4e19-8eff-4035-8238-ae81963b854f, 362,052 bp. (5) c6f29ca1-d84d-4881-9042-dfb37bc9f111, 482,907 bp. (6) 1ccd919f-5726-4d79-8cfe-fe2b344070a1, 275,718 bp. (7) e39308c6-0c73-45d5-9b8d-7f764af858be, 351,045 bp. (8) 86ac29ba-5a93-4c08-aa18-c07829a5b696, 393,007 bp. (9) 64d464d1-f317-4dff-a259-de6097a5cd4c, 221,510 bp. (10) 08e000a1-69dd-40fb-9fd1-942f159ec6b7, 262,585 bp. (11) 1ef64f71-9477-4a5b-bf7e-a356785cc656, 421,096 bp. (12) a1e01c13-7ca1-4dc5-85b1-6b69ec2124f9, 371,129 bp.

**Extended Data Fig. 6:**
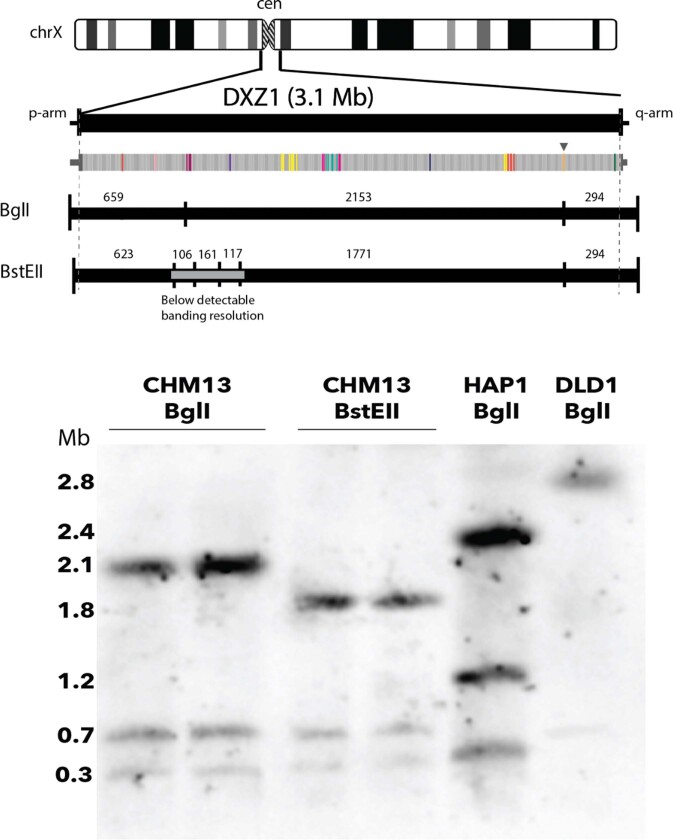
DXZ1 array evaluation by PFGE Southern blotting. Alpha satellite array sizes were estimated by PFGE and Southern blotting using established methods^25,67^. In silico digest of the approximately 3.1-Mb DXZ1 array is predicted to produce three bands with a complete BglI digest: about 659 kb, about 2,153 kb and about 294 kb, which are concordant with the replicate PFGE Southern experiments shown for BglI (about 2.1, about 0.7 and about 0.3 Mb). In silico digest with BstEII provides evidence for six bands, of which three are less than approximately 200 kb and below the range of detection (as marked with grey band). The three remaining bands are once again concordant with observed PFGE-Southern replicates for BstEII (about 1.8, about 0.7 and about 0.3 Mb). HAP1 and DLD1 are included as internal controls. This experiment was repeated seven times with similar results.

Previous high-resolution studies of the haploid centromeric satellite array on the X chromosome (DXZ1) have informed our present genomic models of human centromere organization^8^. The X centromere, as with all normal human centromeres, is defined at the sequence level by alpha satellite DNA—an AT-rich (around 171 bp) tandem repeat, or ‘monomer’^27^. The canonical repeat of the DXZ1 array is defined by 12 divergent monomers that are ordered to form a larger repeating unit of around 2 kb, which is known as a ‘higher-order repeat’ (HOR)^28,29^. The HORs are tandemly arranged into a large, multi-megabase-sized satellite array (that is, 2.2–3.7 Mb; mean of 3,010 kb (s.d. = 429, *n* = 49))^25^ with limited nucleotide differences between repeat copies^8,30,31^. These previous assessments were used to guide our evaluation of the DXZ1 assembly, and offered established experimental methods to evaluate the structure of the DXZ1 array^25,32^ (Extended Data Fig. 5a). To assemble the X centromere, we constructed a catalogue of structural and single-nucleotide variants within the canonical DXZ1 repeat unit (around 2 kb)^28,33^ and used these variants as signposts^8^ to uniquely tile ultra-long reads across the entire centromeric satellite array (DXZ1) (Extended Data Fig. 5b–e), as was previously done for the Y centromere^34^. The DXZ1 array was estimated by pulsed-field gel electrophoresis (PFGE) Southern blotting to be in the range of approximately 2.8–3.1 Mb (Fig. 2b, Extended Data Fig. 6), in which the resulting restriction profiles were in agreement with the structure of the predicted array assembly (Fig. 2 a, b). Copy-number estimates of the DXZ1 repeat by ddPCR were benchmarked against a panel of previously sized arrays by PFGE Southern blotting, and provided further support for an array of around 2.8 Mb (1,408 ± 81.38) copies of the canonical 2,057-kb repeat) (Fig. 2c, Supplementary Table 3, Supplementary Note 7). Furthermore, direct comparisons of DXZ1 structural-variant frequency with PacBio HiFi data were highly concordant^22^ (Fig. 2d, Extended Data Fig. 5c).

**Extended Data Fig. 7:**
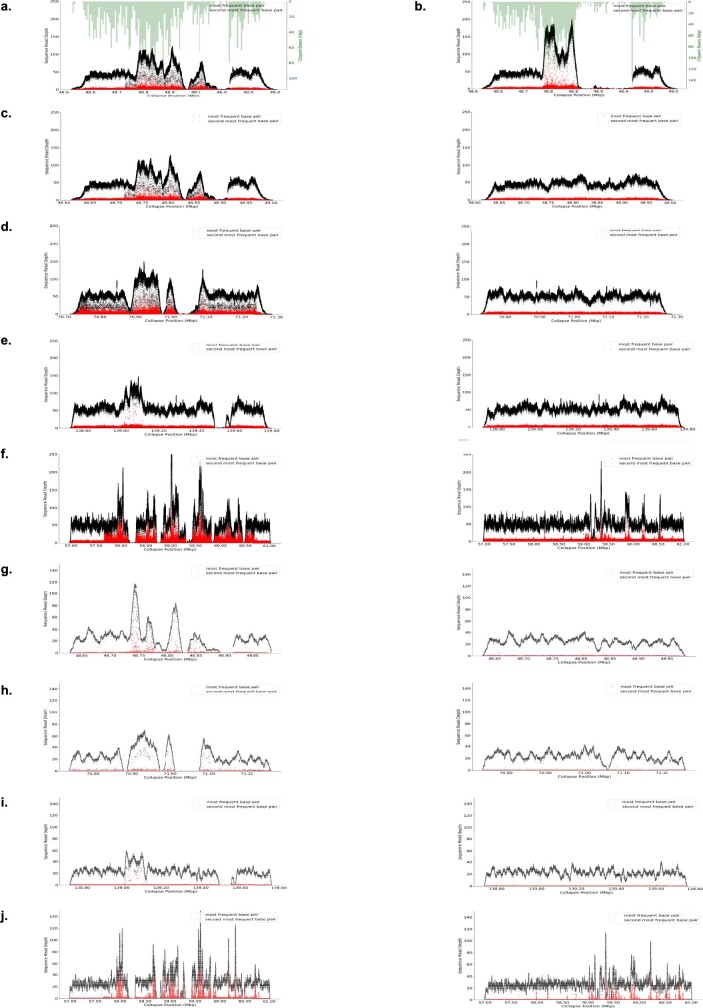
Initial polishing decreased the assembly quality within the largest repeats. **a**, **b**, The initial Canu assembly of the GAGE locus (**a**) was further corrupted owing to standard long-read polishing (arrow, nanopolish) (**b**). Black dots are coverage of the primary allele and red dots are coverage of the secondary allele (PacBio CLR data). The CHM13 genome is effectively haploid so one allele is expected. Regions of low coverage or increased secondary allele frequency indicate low-quality regions or potential repeat collapses. Owing to mismapping of reads during the polishing process, allele coverage becomes less uniform. A modified polishing process, using the unique *k*-mer strategy, corrects this effect. **c**–**f**, The left-side plots are assemblies before polishing. The right-side plots show the same regions after unique *k*-mer-assisted polishing (racon, 2 rounds nanopolish, 2 rounds arrow, 2 rounds 10X). The regions are GAGE locus (48.6–49 Mb) (**c**), 70.8–71.3 Mb (**d**), 138.6–139.7 Mb (**e**) and cenX (57–61 Mb) (**f**). **g**–**j**, Same loci as **c**–**f** but with PacBio HiFi rather than CLR mapped.

**Extended Data Fig. 8:**
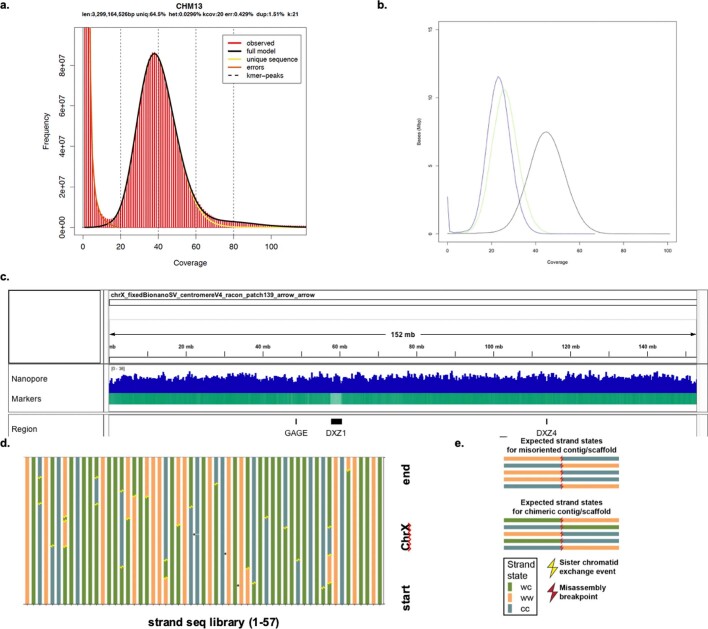
Marker-assisted mapping using unique (single-copy) sequences that are present on the CHM13 X chromosome improve polishing. **a**, 21-mer distribution from the 10X Genomics reads. 21-mers were collected with Meryl and the plot was generated with GenomeScope1.0 to visualize and confirm the haploid nature of CHM13 and genome size (len). k-mers with counts between 5 and 58 (inclusive) were used as unique markers when polishing the X chromosome. **b**, Coverage histograms of PacBio CLR (black), HiFi (blue), and ultra-long (green) reads across the complete X chromosome. Reads were filtered using the same unique marker based filtering as for polishing. **c**, Mapped nanopore reads show uniform coverage across the complete X chromosome. Reads were filtered using the same unique marker based filtering as for polishing. Marker density is shown below the read alignments. **d**, Strand-seq validation of the chromosome X assembly. Strand-seq sequences only single template strands from each homologous chromosome. Sequencing reads originating from such single stranded DNA possess directionality, a feature that can be used to assess a long range contiguity of individual homologues. On the basis of the inheritance of single stranded DNA we distinguish three possible strand states: WW – both homologues inherited Watson template strand, CC – both homologues inherited Watson template strand and WC – one homologue inherited Watson and the other Crick template strand. By tracking changes in strand states along each chromosome we are able to pinpoint locations of recurrent strand state changes that are indicative of a genome misassembly. We have analysed in total 57 Strand-seq libraries and mapped 28 localized strand state changes. These strand state changes are randomly distributed along chromosome X assembly and therefore are indicative of a double-strand break that occurred during DNA replication instead of real genome misassembly. Such breaks are usually repaired by available sister chromatids and therefore often result in change in strand directionality. Black asterisks show small localized strand state changes. Such events are either caused by noisy reads inherent to Strand-seq library preparation or two double-strand-breaks that occurred very close to each other. **e**, Because it is unlikely for a double-strand-break to occur at exactly the same position in multiple single cells, a real genome misassembly is visible in Strand-seq data as a recurrent change in strand state at the same position in a given contig or scaffold. None of these signatures was observed in the CHM13 chromosome X assembly.

Current long-read assemblies require rigorous consensus polishing to achieve maximum base call accuracy^35,36^. Given the placement of each read in the assembly, these polishing tools statistically model the underlying signal data to make accurate predictions for each sequenced base. Key to this process is the correct placement of each read that will contribute to the polishing. Owing to ambiguous read mappings, our initial polishing attempts decreased the assembly quality within the largest X-chromosome repeats (Extended Data Fig. 7a, b). To overcome this, we analysed Illumina sequencing data to catalogue short (21 bp), unique (single-copy) sequences that are present on the CHM13 X chromosome (Extended Data Fig. 8a). Even within the largest repeat arrays, such as DXZ1, there was enough variation between repeat copies to induce unique 21-mer markers at semi-regular intervals (Fig. 2e, f, Extended Data Fig. 8c). These markers were used to inform the correct placement of long X-chromosome reads within the assembly (Methods). Two rounds of iterative polishing were performed for each technology; first with Oxford Nanopore, then PacBio and finally Illumina linked reads^37^, and the consensus accuracy increased after each round. The Illumina data were too short to confidently anchor using unique markers and were only used to polish the unique regions for which mappings were unambiguous. This careful polishing process proved critical for accurately finishing X-chromosome repeats that exceeded both Nanopore and PacBio read lengths.

Our manually finished X-chromosome assembly is complete, gapless and estimated to be 99.991% accurate on the basis of X-specific BACs or 99.995% accurate on the basis of the mapped Illumina data. There is unambiguous support for 99.9% of the assembly bases (Supplementary Note 4), which meets the original Bermuda Standards for finished genomic sequences^38^. Accuracy is predicted to be slightly lower (median identity 99.3%) across the largest repeats, such as the DXZ1 satellite array, but this is difficult to measure owing to a lack of BAC clones from these regions. Mapped long-read and optical-mapping data show uniform coverage across the completed X chromosome and no evidence of structural errors in regions that could be mapped (Fig. 2e, Extended Data Fig. 8b, c, Supplementary Note 4), and Strand-seq data confirm the absence of any inversion errors^39,40^(Extended Data Fig. 8d, e). Single-nucleotide-variant calling through long-read mapping revealed that the initial assembly quality was lower in the large, tandemly repeated GAGE and CT47 gene families, but these issues were resolved by polishing and validated through ultra-long-read mapping and optical mapping (Fig. 1c, d, Extended Data Fig. 7c–j, Supplementary Table 4). Mapped long-read coverage across the DXZ1 array shows uniform depth of coverage and high accuracy, as measured by TandemQUAST^41^ (Fig. 2 e, f, Extended Data Figs. 7j, 8 c). We identified all HiFi reads that match the DXZ1 repeat. All reads—except one with a large, probably erroneous homopolymer—were explained by our reconstruction, confirming the completeness of the DXZ1 array. Mapped coverage across the entire X chromosome was uniform, with coverage of only a small percentage of bases being more than three standard deviations from the mean (0.44% Nanopore, 0.77% PacBio continuous long reads (CLR), 2.4% HiFi). Low-coverage HiFi regions were enriched for low unique-marker density, making them difficult to assign owing to their relatively short length (Supplementary Note 4). Furthermore, variant calling identified no high-frequency variants from the HiFi or CLR data and only low-complexity variants from the ultra-long-read data, which are likely to represent errors in the ultra-long-read data rather than true assembly error. Our complete telomere-to-telomere version of the X chromosome fully resolved 29 reference gaps^3^, totalling 1,147,861 bp of previous ambiguous bases (N-bases).

## Chromosome-wide DNA methylation maps

**Fig. 3:**
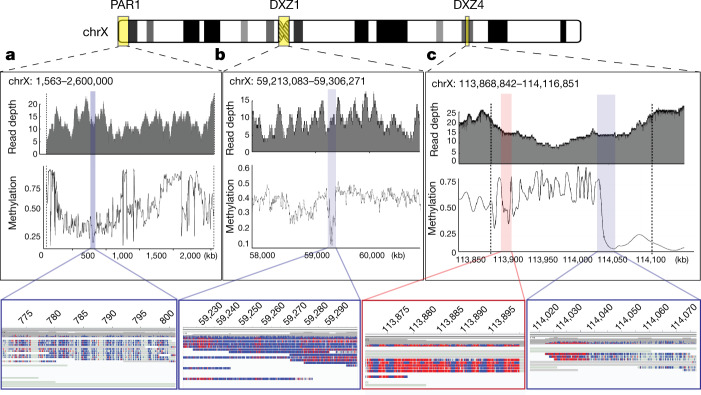
Chromosome-wide analysis of CpG methylation. Methylation estimates were calculated by smoothing methylation frequency data with a window size of 500 nucleotides. Coverage depth and high quality methylation calls (|log-likelihood| > 2.5) for PAR1, DXZ1 and DXZ4 are shown as insets. Only reads with a confident unique anchor mapping and the presence of at least one high-quality methylation call were considered. **a**, Nanopore coverage and methylation calls for pseudoautosomal region 1 (PAR1) of chromosome X (1,563–2,600,000). Bottom Integrated Genomics Viewer (IGV) inset shows a region of hypomethylation within PAR1 (770,545–801,293) with unmethylated bases in blue and methylated bases in red. **b**, Methylation in the DXZ1 array, with bottom IGV inset showing an approximately 93-kb region of hypomethylation near the centromere of chromosome X (59,213,083–59,306,271). **c**, Vertical black dashed lines indicate the beginning and end coordinates of the DXZ4 array. Left IGV inset shows a methylated region of DXZ4 in chromosome X (113,870,751–113,901,499); right IGV inset shows a transition from a methylated to an unmethylated region of DXZ4 (114,015,971–114,077,699).

**Extended Data Fig. 9:**
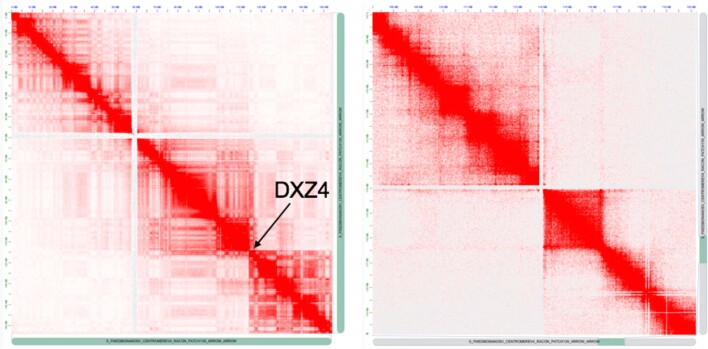
Hi-C read mapping to the chromosome X assembly. The whole X is shown on the left, and the right is zoomed on the DXZ4 locus. The heat map shows clear boundaries around DXZ4, indicating two large superdomains separated by DXZ4.

**Extended Data Fig. 10:**
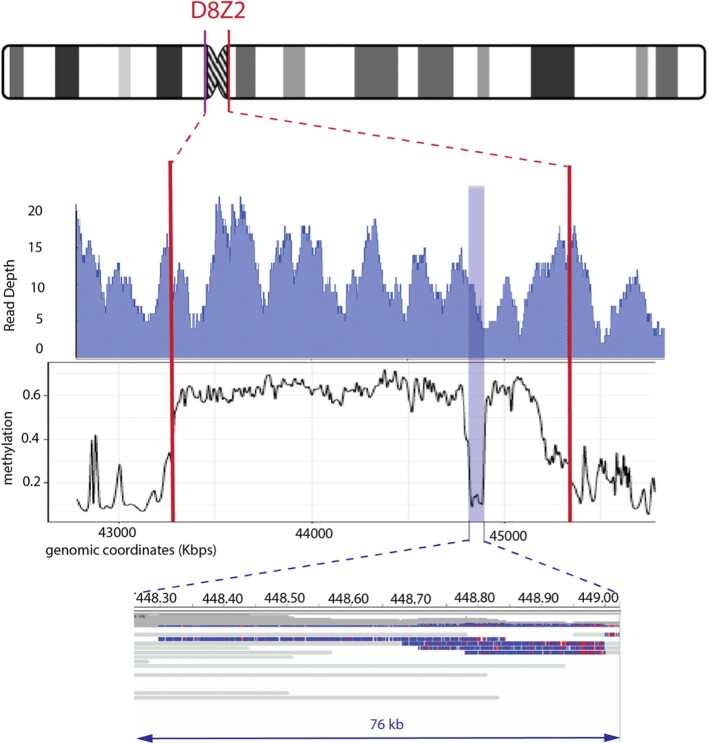
Methylation estimates across centromeric satellite array assembly on chromosome 8 (D8Z2) (chr8: 43,281,085–45,333,062). Methylated values were calculated by smoothing frequency data with a window size of 500 nucleotides. Read coverage shown relies on our unique-anchor mapping and the presence of at least one high-quality methylation call on the read |log-likelihood| > 2.5. Similar to our previous methylation analysis on chromosome X centromeric satellite array (DXZ1), we observe an unmethylated region (about 75 kb) in the centromere of chromosome 8 (as shown: chr8: 44,830,000–44,900,000).

Nanopore sequencing is sensitive to methylated bases, as revealed by modulation in the raw electrical signal^42^. Precisely anchored ultra-long reads provide a new method to profile patterns of methylation over repetitive regions that are often difficult to detect with short-read sequencing. The X chromosome has many epigenomic features that are unique in the human genome. X-chromosome inactivation, in which one of the female X chromosomes is silenced early in development and remains inactive in somatic tissues, is expected to provide a unique methylation profile chromosome-wide. In agreement with previous studies^43^, we observe decreased methylation across the majority of the pseudoautosomal regions (PAR1 and PAR2) located at both tips of the X-chromosome arms (Fig. 3a). The inactive X chromosome also adopts an unusual spatial conformation and, consistent with previous studies^44,45^, CHM13 chromosome conformation capture (Hi-C) data support two large superdomains partitioned at the macrosatellite repeat DXZ4 (Extended Data Fig. 9). On closer analysis of the DXZ4 array we found distinct bands of methylation (Fig. 3c), with hypomethylation observed at the distal edge, which is generally concordant with previously described chromatin structure^46^. Notably, we also identified a region of decreased methylation within the DXZ1 centromeric array (around 60 kb, chrX: 59,217,708–59,279,205) (Fig. 3b). To test whether this finding was specific to the X array or also found at other centromeric satellites, we manually assembled a centromeric array of around 2.02 Mb on chromosome 8 (D8Z2)^47,48^ and used the same unique-marker mapping strategy to confidently anchor long reads across the array (G.A.L. et al., manuscript in preparation). In doing so, we identified another hypomethylated region within the D8Z2 array, similar to our observation on the DXZ1 array (Extended Data Fig. 10)—which further demonstrates the capability of our ultra-long-read mapping strategy to provide base-level chromosome-wide DNA methylation maps. Studies will be needed to validate this finding for additional chromosomes and samples, and to evaluate the potential importance, if any, of these methylation patterns.

## A path for finishing the human genome

This complete telomere-to-telomere assembly of a human chromosome demonstrates that it may now be possible to finish the entire human genome using available technologies. Although we have focused here on finishing the X chromosome, our whole-genome assembly has reconstructed several other chromosomes with only a few remaining gaps, and can serve as the basis for completing additional chromosomes. However, there are still a number of challenges to be overcome. For example, applying these approaches to diploid samples will require phasing the underlying haplotypes to avoid mixing regions of complex structural variation. Our preliminary analysis of other chromosomes shows that regions of duplication and centromeric satellites larger than that of the X chromosome will require the development of additional methods^49^. This is especially true of the acrocentric human chromosomes, the massive satellite arrays and segmental duplications of which have yet to be resolved at the sequence level. In addition, Fig. 1 highlights the centromeric satellite arrays that are expected to be similar in sequence between non-homologous chromosomes. Arrays such as these will need to be phased both between and within chromosomes.

Finishing the human genome will proceed as these remaining challenges are met, beginning with the comparatively easier-to-assemble chromosomes (for example, 3, 6, 8, 10, 11, 12, 17, 18 and 20), and eventually concluding with the chromosomes that contain large blocks of classical human satellites (1, 9 and 16) and the acrocentric chromosomes (13, 14, 15, 21 and 22). In the near term, reference gaps closed in the CHM13 genome will be integrated into GRCh38 using the existing ‘patch’ infrastructure of the GRC. Once all CHM13 chromosomes are completed, we plan to provide these to the GRC as the basis for a new, entirely gapless, reference genome release, which would probably be a mosaic of the current reference with CHM13 sequence in the most difficult regions. Efforts to finally complete the GRC human reference genome will help to advance the necessary technology towards our ultimate goal of complete, telomere-to-telomere, diploid assemblies for all human genomes.

## Methods

### Data reporting

No statistical methods were used to predetermine sample size. The experiments were not randomized and the investigators were not blinded to allocation during experiments and outcome assessment.

### Cell culture

Cells from the complete hydatidiform mole CHM13 were originally cultured from one case of a hydatidiform mole at Magee-Womens Hospital (Pittsburgh) as part of a research study that occurred in the early 2000s (IRB MWH-20-054). At that time, the CHM13 cells were cultured, karyotyped using Q banding, and subsequently immortalized using human telomerase reverse transcriptase (hTERT). For this study, cryopreserved CHM13 cells were thawed and cultured in complete AmnioMax C-100 Basal Medium (Thermo Fisher Scientific) supplemented with 1% penicillin–streptomycin (Thermo Fisher Scientific) and grown in a humidity-controlled environment at 37 °C, with 95% O_2_ and 5% CO_2_. Fresh medium was exchanged every three days and all cells used for this study did not exceed passage 10. Cells have been authenticated and tested negative for mycoplasma contamination.

### Karyotyping

Metaphase slide preparations were made from the human hydatidiform mole cell line CHM13, and prepared by a standard air-drying technique as previously described^51^. DAPI banding techniques were performed to identify structural and numerical chromosome aberrations in the karyotypes according to the ISCN^52^. Karyotypes were analysed using a Zeiss M2 fluorescence microscope and Applied Spectral Imaging software (Supplementary Note 1).

### DNA extraction, library preparation and sequencing

High-molecular-weight DNA was extracted from 5 × 10^7^ CHM13 cells using a modified Sambrook and Russell protocol^1,53^. Libraries were constructed using the Rapid Sequencing Kit (SQK-RAD004) from Oxford Nanopore Technologies with 15 μg of DNA. The initial reaction was typically divided into thirds for loading and FRA buffer (104 mM Tris pH 8.0, 233 mM NaCl) was added to bring the volume to 21 ul. These reactions were incubated at 4 °C for 48 h to allow the buffers to equilibrate before loading. Most sequencing was performed on the Nanopore GridION with FLO-MIN106 or FLO-MIN106D R9 flow cells, with the exception of one Flongle flow cell used for testing. Sequencing reads used in the initial assembly were first base-called on the sequencing instrument. After all data were collected, the reads were base-called again using the more recent Guppy algorithm (v.2.3.1 with the ‘flip-flop’ model enabled).

A 10X Genomics linked-read genomic library was prepared from 1 ng of high-molecular-weight genomic DNA using a 10X Genomics Chromium device and Chromium Reagent Kit v.2 according to the manufacturer’s protocol. The library was sequenced on an Illumina NovaSeq 6000 DNA sequencer on an S4 flow cell, generating 586 million paired-end 151-base reads. The raw data were processed using RTA 3.3.3 and bwa 0.7.12^54^. The resulting molecule size was calculated to be 130.6 kb from a Supernova^55^ assembly.

DNA was prepared using the ‘Bionano Prep Cell Culture DNA Isolation Protocol’. After cells were collected, they were put through a number of washes before embedding in agarose. A proteinase K digestion was performed, followed by additional washes and agarose digestion. The DNA was assessed for quantity and quality using a Qubit dsDNA BR Assay kit and CHEF gel. A 750-ng aliquot of DNA was labelled and stained following the Bionano Prep Direct Label and Stain (DLS) protocol. Once stained, the DNA was quantified using a Qubit dsDNA HS Assay kit and run on the Saphyr chip.

Hi-C libraries were generated, in replicate, by Arima Genomics using four restriction enzymes. After the modified chromatin digestion, digested ends were labelled, proximally ligated, and then proximally ligated DNA was purified. After the Arima-HiC protocol, Illumina-compatible sequencing libraries were prepared by first shearing then size-selecting DNA fragments using SPRI beads. The size-selected fragments containing ligation junctions were enriched using Enrichment Beads provided in the Arima-HiC kit, and converted into Illumina-compatible sequencing libraries using the Swift Accel-NGS 2S Plus kit (P/N: 21024) reagents. After adaptor ligation, DNA was PCR-amplified and purified using SPRI beads. The purified DNA underwent standard quality control (qPCR and Bioanalyzer) and was sequenced on the HiSeq X following the manufacturer’s protocols.

### Nanopore and PacBio whole-genome assembly

Canu v.1.7.1^21^ was run with all rel1 Oxford Nanopore data (on-instrument basecaller, rel1) generated on or before 7 November 2018 (totalling 39× coverage) and PacBio sequences (Sequence Read Archive (SRA): PRJNA269593) generated in 2014 and 2015 (totalling 70× coverage)^2,56^. Several chromosomes in the assembly are broken only at centromeric regions (for example, chr10, chr12, chr18 and so on) (Fig. 1). Despite apparent continuity across several centromeres, (for example, chr8, chr11 and chrX), the assembler reported many fewer than the expected number of repeat copies.

### Manual gap closure

Gaps on the X chromosome were closed by mapping all reads against the assembly and manually identifying reads joining contigs that were not included in the automated Canu assembly. This generated an initial candidate chromosome assembly, with the exception of the centromere. Four regions of the candidate assembly were found to be structurally inconsistent with the Bionano optical map and were corrected by manually selecting reads from those regions and locally reassembling with Canu^21^ and Flye v.2.4^57^. Low-coverage long reads that confidently spanned the entire repeat region were used to guide and evaluate the final assembly where available. Evaluation of copy number and repeat organization between the reassembled version and spanning reads was performed using HMMER (v.3)^58,59^ trained on a specific tandem repeat unit, and the reported structures were manually compared. Default parameters for Minimap2^60^ resulted in uneven coverage and polishing accuracy over tandemly repeated sequences. This was successfully addressed by increasing the Minimap2 -r parameter from 500 to 10,000 and increasing the maximum number of reported secondary alignments (-N) from 5 to 50. Final evaluation of repeat base-level quality was determined by mapping of PacBio datasets (CLR and HiFi) (Extended Data Fig. 7, Supplementary Note 4).

The alpha satellite array in the X centromere, owing to its availability as a haploid array in male genomes, is one of the best-studied centromeric regions at the genomic level, with a well-defined 2-kb repeat unit^28^, physical and genetic maps^8,30^ and an expected range of array lengths^25^. We initially generated a database of alpha satellite containing ultra-long reads, by labelling those reads with at least one complete consensus sequence^33^ of a 171-bp canonical repeat in both orientations, as previously described^61^. Reads containing alpha in the reverse orientation were reverse-complemented, and screened with HMMER (v.3) using a 2,057-bp DXZ1 repeat unit. We then used run-length encoding in which runs of the 2,057-bp canonical repeat (defined as any repeat in the range of minimum: 1,957 bp, maximum: 2,157 bp) were stored as a single data value and count, rather than the original run. This allowed us to redefine all reads as a series of variants, or repeats, that differ in size or structure from the expected canonical repeat unit, with a defined spacing in between. Identified CHM13 DXZ1 structural variants in the ultra-long-read data were compared to a library of previously characterized rearrangements in published PacBio (CLR^50^ and HiFi^22^) using Alpha-CENTAURI, as described^61^. Output annotation of structural variants and canonical DXZ1 spacing for each read were manually clustered to generate six initial contigs, two of which are known to anchor into the adjacent Xp or Xq. To define the order and overlap between contigs, we identified all 21-mers that had an exact match within the high-quality DXZ1 array data obtained from CRISPR–Cas9 Duplex-seq (CRISPR-DS) targeted resequencing^62^ (Supplementary Note 8). Overlap between the two or more 21-mers with equal spacing guided the organization of the assembly. Orthogonal validation of the spacing between contigs (and contig structure) was supported with additional ultra-long read coverage, providing high-confidence in repeat unit counts for all but three regions.

### Chromosome X long-read polishing

We used a novel mapping pipeline to place reads within repeats using unique markers. Length *k* substrings (*k*-mers) were collected from the Illumina linked reads, after trimming off the barcodes (the first 23 bases of the first read in a pair). The read was placed in the location of the assembly that had the most unique markers in common with the read. Alignments were further filtered to exclude short and low-identity alignments. This process was repeated after each polishing round, with new unique markers and alignments recomputed after each round. Polishing proceeded with one round of Racon followed by two rounds of Nanopolish and two rounds of Arrow. Post-polishing, all previously flagged low-quality loci showed substantial improvement, with the exception of 139–140.3 which still had a coverage drop and was replaced with an alternate patch assembly generated by Canu using PacBio HiFi data.

### Whole-genome long-read polishing

The rest of the whole-genome assembly was polished similarly to the X chromosome, but without the use of unique *k*-mer anchoring. Instead, two rounds of Nanopolish, followed by two rounds of Arrow, were run using the above parameters, which rely on the mapping quality and length and identity thresholds to determine the best placements of the long reads. As no concerted effort was made to correctly assemble the large satellite arrays on chromosomes other than the X chromosome, this default polishing method was deemed sufficient for the remainder of the genome. However, future efforts to complete these remaining chromosomes are expected to benefit from the unique *k*-mer anchoring mapping approach.

### Whole-genome short-read polishing

The Illumina linked reads were used for a final polishing of the whole assembly, including the X chromosome, but using only unambiguous mappings and correcting only small insertion and deletion errors (Supplementary Note 4).

### Methylation analysis

To measure CpG methylation in nanopore data we used Nanopolish^63^. Nanopolish uses a Hidden Markov model on the nanopore current signal to distinguish 5-methylcytosine from unmethylated cytosine. The methylation caller generates a log-likelihood value for the ratio of probability of methylated to unmethylated CGs at a specific *k*-mer. We next filtered methylation calls using the nanopore_methylation_utilities tool (https://github.com/isaclee/nanopore-methylation-utilities), which uses a log-likelihood ratio of 2.5 as a threshold for calling methylation^64^. CpG sites with log-likelihood ratios greater than 2.5 (methylated) or less than −2.5 (unmethylated) are considered high quality and included in the analysis. Reads that did not have any high-quality CpG sites were excluded from the subsequent methylation analysis. Figure 3 shows the coverage of reads with at least one high quality CpG site. Nanopore_methylation_utilities integrates methylation information into the alignment BAM file for viewing in the bisulfite mode in IGV^65^ and also creates Bismark-style files which we then analysed with the R Bioconductor package BSseq (v.1.20.0)^66^. We used the BSmooth algorithm^66^ within the BSseq package for smoothing the data to estimate the methylation level at specific regions of interest.

### Reporting summary

Further information on research design is available in the Nature Research Reporting Summary linked to this paper.

## Online content

Any methods, additional references, Nature Research reporting summaries, source data, extended data, supplementary information, acknowledgements, peer review information; details of author contributions and competing interests; and statements of data and code availability are available at 10.1038/s41586-020-2547-7.

## Supplementary information

Supplementary Information This file contains Supplementary Notes 1-8, which detail analyses from the main text, Supplementary Table 1 that provides genome annotation results, Supplementary Table 2 that provides inversion calls, Supplementary Table 3, which provides a description of all human genome assemblies in NCBI with contig NG50 >25 Mb or originating from CHM13; Supplementary Table 4 provides DXZ1 array estimates, Supplementary Table 5 lists structural variants identified by BioNano optical maps, and additional references (see Contents for more details).

Reporting Summary

## Data Availability

Original data generated at the Stowers Institute for Medical Research that underlie this manuscript can be accessed from the Stowers Original Data Repository at http://www.stowers.org/research/publications/libpb-1453. Genome assemblies and sequencing data including raw signal files (FAST5), event-level data (FAST5), base calls (FASTQ) and alignments (BAM/CRAM) are available as an Amazon Web Services Open Data set. Instructions for accessing the data, as well as future updates to the raw data and assembly, are available from https://github.com/nanopore-wgs-consortium/chm13. All data are also archived and available under NCBI BioProject accession PRJNA559484, including the whole-genome assembly (GCA_009914755.1) and completed X chromosome (CM020874.1).

## Code availability

No custom code was used for the analysis in this manuscript. All used software is freely available: Canu, https://github.com/marbl/canu; BWA, https://github.com/lh3/bwa; Minimap2, https://github.com/lh3/minimap2; Arrow, https://github.com/PacificBiosciences/GenomicConsensus; Nanopolish, https://github.com/jts/nanopolish; HMMER, http://hmmer.org; Supernova, https://support.10xgenomics.com; Long Ranger, https://support.10xgenomics.com; Juicer & Juicebox, https://github.com/aidenlab/Juicebox; Flye, https://github.com/fenderglass/Flye; Bioconductor, https://github.com/Bioconductor; Samtools, http://samtools.github.io; Freebayes, https://github.com/ekg/freebayes; MUMmer, http://mummer.sourceforge.net; CRISPR-DS, https://github.com/risqueslab.
