# Supplementary material for: Telomere-to-telomere assembly of a complete human X chromosome
Source: Nature. 2020 Jul 14;585(7823):79–84. doi: 10.1038/s41586-020-2547-7 (PMC7484160; doi:10.1038/s41586-020-2547-7)
Supplement: Supplementary file 1 — This file contains Supplementary Notes 1-8, which detail analyses from the main text, Supplementary Table 1 that provides genome annotation results, Supplementary Table 2 that provides inversion calls, Supplementary Table 3, which provides a description of all human genome assemblies in NCBI with contig NG50 >25 Mb or originating from CHM13; Supplementary Table 4 provides DXZ1 array estimates, Supplementary Table 5 lists structural variants identified by BioNano optical maps, and additional references (see Contents for more details). [file 41586_2020_2547_MOESM1_ESM.pdf]

nature research

https://doi.org/10.1038/s41586-020-2547-7

Supplementary information

Telomere-to-telomere assembly of a complete human X chromosome

In the format provided by the authors and unedited

Supplementary Information for
Telomere-to-telomere assembly of a complete human X chromosome

This PDF file includes:
Supplementary Notes 1 to 8
Tables S1 to S5
References

Supplementary Note 1. CHM13 cell line and chromosome characterization

CHM13hTERT Cell Line
CHM13 cells were originally grown in culture from one case of a hydatidiform mole at Magee-Womens Hospital (Pittsburgh, PA) as part of a research study (IRB MWH-20-054). Cryogenically frozen cells from this culture were grown and transformed using human telomerase reverse transcriptase (TERT) to develop a cell line. This cell line retains a 46,XX karyotype and complete homozygosity.

Spectral karyotyping (SKY)
Spectral imaging was performed using In laser-scanning confocal microscopes LSM-710 and LSM-780 (Carl Zeiss Microimaging, Jena, Germany). Both microscopes were equipped with a QUASAR detection unit that can acquire with a single scan an entire range of emission wavelengths (in 10 nm increments) for subsequent spectral unmixing. For spectral imaging, 3 excitation laser lines were utilized: 488, 561, and 633 nm. Images were collected with 3 different dichroics: the first passing 488 nm excitation, the second passing 488 nm and 561 nm excitation, and the third passing all 3 laser lines. In addition, a 405 nm laser was used to acquire a Hoechst 33342– stained DNA image for segmentation, with emission collected at ~450 nm. All images were acquired with either a 40× or 63× Plan Apochromat objective (Carl Zeiss Microimaging). Pinhole settings were optimized for background reduction and signal-to-noise ratio. Image processing and karyotyping of the CHM13 line were performed with a set of custom open source ImageJ (NIH, Bethesda, MD) plugins called Karyotype Identification via Spectral Separation (KISS), freely available at http://research.stowers.org/imagejplugins/KISS_analysis.html. Briefly, the plugins perform interactive background subtraction, spectral unmixing, interactive chromosome segmentation, and interactive karyotyping based on dye composition. Chromosome segmentation is performed using a semi-automated method based on the Hoechst image. First, the image is smoothed with a Gaussian blur with a 1 pixel standard deviation and then segmented with a manually chosen fractional threshold and object area limits to eliminate dirt and intact nuclei. Next, chromosomes too close to be separated by thresholding are manually separated. Finally overlapping chromosomes are separated into non-overlapping parts and then linked together for

karyotyping. A total of 10 SKY images were evaluated to assess the stability of the CHM13 line.

Supplementary Note 2. CHM13 admixture analysis

We ran the software ADMIXTURE v1.3.0[1] with 10-fold cross validation (CV) on a diversity panel of 1,964 unrelated individuals from the 1000 Genomes Project (1KG, 20140818 release)[2] and Simons Genome Diversity Panel (SGDP)[3]. SNVs were called previously and data from the SGDP and 1KG were prepared by left normalizing variants with bcftools v1.9[4] (to standardize indels), followed by filtering for <10% missing genotypes within an individual and across individuals for a given SNP (<50% missing genotypes across a SNP), minor allele frequency (>5%), and LD pruning with PLINK 1.90[5]. Sites were then converted from GRCh37 to GRCh38 using UCSC liftover. This resulted in a total of ~155,000 SNP sites. Based on the smallest CV error, we determine an optimal value for the K parameter (K=9), but also considered other values (K=6 to K=14) since the CV errors were marginally different. Since CHM13 is comprised of only one haplotype, we used the allele frequencies learned by ADMIXTURE from the diversity panel to assess the ancestry of CHM13 in a supervised manner by using ADMIXTURE to project its ancestry. Missing genotypes were assumed to be reference as CHM13 was not jointly-genotyped with the reference samples. We assigned CHM13 to the cluster which contributed the largest proportion of ancestry and then grouped clusters into super populations according to membership of known populations from the reference cohort. We assessed our inference by visualizing the proportion of ancestry from each cluster for a random subset of 10 individuals from each known population as well as CHM13. While this analysis is preliminary and ADMIXTURE is not optimized to handle data from a genome of a single haplotype, the analysis consistently predicted an admixed haplotype predominantly of European ancestries.

Supplementary Note 3. Library preparation and sequencing

Oxford Nanopore

Library preparation and nanopore sequencing was performed as previously described[6], with the following updates. Generation of ultra-long reads employs the Rapid Sequencing Kit (Oxford Nanopore Technologies, UK) and comprises two steps: tagmentation of DNA by a transposase complex followed by attachment of the sequencing adapter. Previous work was performed using kit SQK-RAD002 which was replaced by SQK-RAD003 in Jun 2017. Testing performed on this kit indicated difficulty generating ultra-long reads was due to a protocol change which doubled the standard input required from 200 ng to 400 ng and a reformulation of the FRM reagent (now called FRA). This protocol resulted in low efficiency libraries when using HMW DNA input. Testing showed that reducing the volume of fragmentation reagent from 5 ul to 1.5 ul and the addition of 0.02% Triton-X100 final concentration could restore library performance. The modifications are included in the 'Ultra-long read sequencing protocol for RAD004' (dx.doi.org/10.17504/protocols.io.mrxc57n) used here.
High-molecular-weight genomic DNA from the CHM13hTERT cell line was obtained using a modified Sambrook and Russell DNA extraction method before preparing ultra-long read sequencing libraries using the protocol above. Briefly, 16 μl of DNA from the Sambrook

extraction at approximately 1 µg/µl, manipulated with a wide-bore P20 pipette tip, was placed in a 0.2 ml PCR tube, with 1 µl removed to confirm quantification value. 3.5ul EB and 1.5 µl FRA (SQK-RAD004, ONT) was added and mixed slowly ten times by gentle pipetting with a wide-bore pipette tip moving only 18 µl. After mixing, the sample was incubated at 30 °C for 1 min followed by 80 °C for 1 min on a thermocycler. After this, 1 µl RAP (SQK-RAD004, ONT) was added and mixed slowly ten times by gentle pipetting with a cut-off pipette tip moving only 14 µl. The library was then incubated at room temperature for 30 min to allow adapter attachment. Libraries are divided, diluted and incubated for 48 hours (as discussed in updates above). To load the library, 34 µl SQB (SQK-RAD004, ONT) was mixed with 20 µl nuclease-free water, and this was added to the library. Using a P100 wide-bore tip set to 75 µl, this library was mixed by pipetting slowly five times. This extremely viscous sample was loaded onto the "spot on" port and entered the flow cell by capillary action. The standard loading beads were omitted from this protocol owing to excessive clumping when mixed with the viscous library.

GridION sequencing was performed as per manufacturer's guidelines using R9/R9.4 flow cells (FLO-MIN106 or FLO-MIN106D, ONT), and controlled using Oxford Nanopore Technologies MinKNOW (version 3.4.5) software. The specific versions of the software used varied from run to run but can be determined by inspection of the provided fast5 files. This generated the rel1 dataset.

Reads from all sites were copied to the NIH Biowulf HPC cluster, where base calling was performed using Guppy (flip-flop version 2.3.1) to generate the updated dataset (referred to as rel2).

10x Genomics

A linked read genomic library was prepared from one nanogram of high molecular weight genomic DNA using a 10x Genomics Chromium device and Chromium Reagent Kit v2 according to manufacturer's protocol. The library was sequenced on a NovaSeq 6000 DNA sequencer (Illumina, Inc.) on an S4 flow cell, generating 586M paired-end 151 base reads. The raw data was processed using RTA3.3.3 and bwa0.7.12. The resulting molecule size was calculated to be 130.6 kb from a Supernova assembly.

Bionano optical mapping

DNA was prepared using the 'Bionano Prep Cell Culture DNA Isolation Protocol'. After cells were harvested, they were put through a number of washes before embedding in agarose. A proteinase K digestion was performed, followed by additional washes and agarose digestion. From this point, the DNA was drop dialyzed and allowed to equilibrate at room temperature for two days. The DNA was assessed for quantity and quality using a Qubit dsDNA BR Assay kit and CHEF gel. A 750 ng aliquot of DNA was labeled and stained following the Bionano Prep Direct Label and Stain (DLS) protocol. Once stained, the DNA was quantified using a Qubit dsDNA HS Assay kit and run on the Saphyr chip.

Hi-C sequencing

Hi-C libraries were generated, in replicate, by Arima Genomics using a modified version of the Arima-HiC kit. Briefly, the current Arima-HiC kit (P/N: A510008) utilizes 2 restriction enzymes for simultaneous chromatin digestion. In the modified protocol, 4 restriction enzymes were deployed to enable more uniform per base coverage of the genome while maintaining the highest long-range contiguity signal, thereby benefiting analyses such as base polishing, scaffolding, and phasing. After the modified chromatin digestion, digested ends were labelled, proximally ligated, and then proximally-ligated DNA was purified. After the Arima-HiC protocol, Illumina-compatible sequencing libraries were prepared by first shearing purified Arima-HiC proximally-ligated DNA and then size-selecting DNA fragments using SPRI beads. The size-selected fragments containing ligation junctions were enriched using Enrichment Beads provided in the Arima-HiC kit, and converted into Illumina-compatible sequencing libraries using the Swift Accel-NGS 2S Plus kit (P/N: 21024) reagents. After adapter ligation, DNA was PCR amplified and purified using SPRI beads. The purified DNA underwent standard QC (qPCR and Bioanalyzer) and sequenced on the HiSeq X following manufacturer's protocols.

Supplementary Note 4. Assembly and chromosome X finishing

Nanopore and PacBio whole-genome assembly

Canu 1.7.1 was used for analysis with the parameters `genomeSize=3.1g corMhapSensitivity=normal ovlMerThreshold=500 correctedErrorRate=0.085 trimReadsCoverage=2 trimReadsOverlap=500 -pacbio-raw` for both data types (Nanopore and PacBio). The X was selected for finishing based on an earlier assembly using the same PacBio data but including only Oxford Nanopore data generated on or before 2018/08/29. Reads were mapped to the assembly using Minimap2 with parameters `-ax map-ont` to identify those spanning gaps and not included in the assembly. The X chromosome, excluding the centromere, was polished using one round of Medaka using only reads assigned by the assembler to the X chromosome. Arrow[7] was run using the ArrowGrid pipeline available at https://github.com/skoren/ArrowGrid using only the P6-C4 chemistry data[8] listed here: https://github.com/nanopore-wgs-consortium/CHM13. The default alignment identity was changed from 0.75 to 0.85. The full assembly, excluding the X centromere, was polished using Nanopolish v0.11.0 using the pipeline available at https://github.com/skoren/NanoGrid. Reads were mapped using Minimap2 with the options `-ax map-ont`. Nanopolish used options `variants --methylation-aware=cpg --consensus -min-candidate-frequency 0.01 --fix-homopolymers`. Arrow v2.2.2 from SMRTlink 6.0.0.47841 was run on the full assembly, again excluding the centromere, with the mapping identity increased to 0.85 `--minAccuracy=0.85 --minLength=5000 --minAnchorSize=12 --maxDivergence=30 --concordant --algorithm=blasr --algorithmOptions=--useQuality --maxHits=1 --hitPolicy=random --seed=1` and additional parameters `-x 10 -q 0 -X120 -v --algorithm=arrow`.

10x Genomics whole-genome assembly and validation

The 10x data was assembled with Supernova v2.1.1 using the command `run --maxreads=all --id=CHM13 --fastqs=Chromium`. This resulted in a 2.95 Gbp assembly with a contig NG50 of 209.7 kbp and scaffold NG50 of 38.5 Mbp for pseudohaplotype 1 and a 2.95 Gbp assembly with a contig NG50 of 209.7 kbp and scaffold NG50 of 38.5 Mbp for pseudohaplotype 2.
Prior to optical mapping, 10x Genomics / Illumina data was mapped to the full assembly using Long Ranger v2.2.2 with the options `longranger align --jobmode=slurm --localcores=32 --localmem=60 --maxjobs=500 --jobinterval=5000 --disable-ui --nopreflight`. Any regions with ≥10-fold coverage were marked as supported. Adjacent supported regions were merged if they were within 500 bp of each other. This list of supported regions was inverted and any unsupported regions within 2 kbp of each other were merged. Finally, the assembly was split at any low-coverage region ≥50 kbp.

Bionano optical map assembly and scaffolding
The raw data was assembled with the Bionano Solve data analysis software. This software generated whole genome map assemblies, along with alignments to the reference sequences. In this case, the CHM13 assembly was aligned with CHM13 optical map. After breaking potential mis-assemblies identified by the 10x data, hybrid scaffolding was run using the optical map data using the command `hybridScaffold.pl -n $ASM -b DLE1.cmap -c hybridScaffold_DLE1_config.xml -r avx/RefAligner -B 2 -N 2 -f -o $PWD/scaffold`.

Hi-C analysis
Hi-C read mapping heatmap was generated using Juicer v1.5.6 available from https://github.com/VGP/vgp-assembly/tree/master/pipeline/juicer. The restriction site position was indexed with `python juicer-1.5.6/misc/generate_site_positions.py MboI asm asm.fasta` and .hic files were generated with default options ``juicer.sh -z `pwd`/reference/asm.fasta -y `pwd`/reference/asm_MboI.txt -D /usr/local/apps/juicer/juicer-1.5.6/ -d `pwd` -p `pwd`/reference/chr.sizes``. The maps were visualized with Juicebox v1.8.8.

Assembly chromosome assignment
The final scaffolds were assigned to chromosomes by NCBI. Briefly, the automated assembly alignment pipeline was used to build a list of scaffold to chromosome mappings. This list was manually reviewed to assign additional scaffolds or re-assign scaffolds to chr Un as necessary. Based on this assignment, there are 6 chromosomes with 90% of their length covered by 2 or fewer contigs (3, 6, 10, 12, 18, X) and 10 chromosomes (3, 5, 6, 8, 10, 12, 17, 18, 20, X) covered by 2 or fewer scaffolds.

Telomere Analysis
We re-used the telomere identification scripts from the vertebrate genome project (https://github.com/VGP/vgp-assembly/tree/master/pipeline/telomere). Windows of 1000 bp where at least 50% of the sequence matches the canonical vertebrate telomere (TTAGGG) in

either strand were identified. Overlapping windows were merged and windows within 5kb of the scaffold end marked as putative telomeres. The X chromosome contig had two telomeres at both ends, as expected. The first from 0–1800 bp and and the second from 154,267,200–154,268,800 bp. In total, we identified 41 of 46 expected telomeres in the assembly. The chromosomes with two telomeres on assigned scaffolds are: 1, 2, 4, 6, 7, 8, 11, 12, 16, 17, 20, and X. Chromosomes 3, 5, 9, 10, 13, 14, 15, 18, 19, 21 have one telomere on an assigned scaffold, and chromosome 22 has none. The remaining seven telomere arrays were in short contigs which we could not assign to a chromosome. The average telomere length in the assembly is 3,215 bp.

We used the same strategy to identify telomeres in ONT reads >50 kbp and all HiFi PacBio reads. Reads with a telomere within 5 kbp of the read start or end are marked as telomeric reads. Telomere lengths (based on the merged overlapping windows) in both the HiFi (mean=2,448 bp) and ONT (mean=2,368 bp) reads are concordant in size with each other (Extended Data Fig4).

Chromosome X validation and fixes

The assembled optical map was used to call high-confidence structural variants on the entire assembly, including the candidate X chromosome. This identified four structural variants (Supplementary Table 4). These SVs were confirmed by discordantly mapping reads later identified in the rel2 ultra-long dataset. To correct these assembly errors, reads over 100 kb with breaks near the variant site were extracted for each SV, making four sets of reads. Each read set was then assembled separately with default parameters by both Canu 1.8 and Flye 2.4[9,10]. The two assemblers had good agreement and the Flye contigs were aligned to the chromosome X draft and used as patches to replace the incorrect sequence in the original assembly. The patched assembly was once again validated by the optical map, which now reported no discrepancies. PacBio HiFi reads were aligned to the X chromosome and potential repeat collapses identified using a previously described method[11]. This analysis identified the GAGE locus (48.7–48.9 Mbp), cenX (57–61 Mbp), 122 kb segmental duplication containing CXorf49 gene copies (69.5–71.2 Mbp), and CT45 (138.6–139.7 Mbp) as regions of potential collapse (Extended Data Fig7). Manual inspection as well as optical map support suggested these regions were not typical repeat collapses, but residual consensus errors due to uneven polishing of large repeat arrays, which were later resolved using a novel polishing strategy as described below.

Chromosome X long-read polishing

Unique *k*-mers were identified as those having a copy number in the Illumina read set roughly equal to the expected depth of coverage (between 5 and 58, Extended Data Fig8a) using Meryl[12] from Canu snapshot v1.8 +298 changes (r9508 aab8e5dc15c6b20addccd809c2cc6a62c1fa9c46). In brief, *k*-mers were counted with `meryl count k=21 output 10x.meryl $FASTQ` and filtered with `meryl greater-than 5 output 10x.gt5.meryl 10x.meryl` and `meryl less-than 58 output 10x.gt5.lt58.meryl 10x.gt5.meryl`. Those *k*-mers having both the expected copy

number in the 10x data and occuring once in the assembled genome were selected as putative unique markers. `meryl equal-to 1 output asm_1.meryl [ count k=21 asm.fasta ]` was run to collect single-copy kmers in the assembly, and it was intersected with `meryl intersect output 10x_asm_single.meryl 10x.gt5.lt58.meryl asm_1.meryl`. Reads were mapped using Minimap2 v2.71-941 with the parameters `-N 50 -r 10000 -ax map-ont`. These parameters increase the number of candidate sites reported for a read and tolerate larger gaps within a read without breaking to better allow correction of larger indels in repeat arrays. The Minimap2 alignments were converted to sequence, replacing any mis-matched or missing bases in the read with Ns, and these sequences were scored using the unique markers and placed in the location maximizing the unique marker matches. This generated a new SAM file with all uniquely placed reads assigned a Phred mapping quality (MQ) value of 60. This SAM was filtered to exclude short CIGAR strings (<50 kb for Nanopore, <10 kb for PacBio), and those below a minimum length / identity threshold (25 kb at 75% identity for Nanopore and 5 kb at 75% identity for PacBio). Racon used the parameters `-w 5000 -e 0.2`. Nanopolish v0.11.0 ran with `minimap2 -ax map-ont -N 50 -r 10000` for mapping and `nanopolish variants --methylation-aware=cpg --consensus --min-candidate-frequency 0.01 --fix-homopolymers` for consensus. Arrow v2.2.2 ran with `minimap2 -ax map-pb -N 50 -r 1000` for mapping and `-x 10 -q 0 -X120 -v --algorithm=arrow` for consensus.

Whole-genome short-read polishing
The 10x data was mapped to the scaffolded and polished assembly using Long Ranger v2.2.2 and the options longranger align `--jobmode=slurm --localcores=32 --localmem=60 --maxjobs=500 --jobinterval=5000 --disable-ui --nopreflight`. FreeBayes v1.2.0 was used to call variants with the command freebayes `-I -F 0.5 -m 50 --min-alternate-total 5 --min-coverage 10 --max-coverage 100 --read-snp-limit 5 --read-mismatch-limit 5`, which enforces a conservative minimum MQ of 50 and only corrects indels supported by more than half of the Illumina reads. This was repeated for two rounds.

Chromosome X mapping and variant identification
The three available long-read technologies (PacBio HiFi, PacBio CLR, Nanopore UL) were mapped to the final chrX contig using the same unique marker based filtering as used for polishing. The coverage distribution matched the expected normal distribution (Extended Data Fig 8b, UL: mean: 26.08, sd: 6.05; CLR: mean: 44.87, sd: 8.66; HiFi: mean: 22.99, sd: 6.27) with a low fraction of bases above or below 3 standard deviations (0.44% for UL, 0.77% for PacBio CLR, 2.24% for PacBio HiFi). The low-coverage HiFi regions were enriched for low frequency of unique markers (mean spacing 3,342 bp vs 66 for the whole X) which we attribute to their relatively short length and lower coverage.

We ran the variant caller Sniffles[13] v1.0.11 with the command:

```
sniffles --genotype -t 16 -m chrX.bam -v chrX.vcf
```

We then counted the number of variants from each data type with allele frequency ≥75%. As

CHM13 is haploid, we expect true errors to have high read support. No variants meeting the threshold were identified in the HiFi or CLR data. 79 variants were identified in the UL data but they were short (mean: 130.0 bp) and enriched for simple sequence repeats or homopolymers (75.93% masked by sdust versus 4.66% in the entire X chromosome) and are likely base calling errors in the UL data.

Assembly quality estimation

We estimated final assembly QV and completeness using previously sequenced CHM13 BACs targeting segmental duplications (VMRC59 library), as well as concordance with the 10x Genomics data. All nucleotide sequences matching VMRC59 with "complete" in the name were downloaded from NCBI. This gave a total of 341 complete BACs. The BACs were mapped with minimap2 with the command `minimap2 --secondary=no -ax asm20 -r 2000` and evaluated using the pipeline available from https://github.com/skoren/bacValidation. For 10x Genomics, both Supernova haplotypes were combined and a BAC was considered resolved if either pseudo-haplotype assembly captured it. Out of these 341 BACs, 280 mapped over 99.5% of their length to our CHM13 assembly, which compares favorably to previous assemblies (main text, Table 1). The identity of all BACs mapping over 99.5% of their length was also high for our assembly at 99.98% (Q37.04) median/99.80% (Q27.05) mean vs 99.98% (Q37.32)/99.72% (Q25.60) for PacBio CLR w/ FALCON + Quiver + Pilon, 99.98% (Q36.86)/99.76% (Q26.25) for PacBio HiFi w/ Canu, 99.97% (Q35.97)/99.86% (Q28.45) for 10x Genomics w/ Supernova, and 99.73% (Q25.70)/99.48% (Q22.87) for GRCh38. Using the 31 unique BACs, the identities increase further to 99.99% (Q42.29) median/99.98% (Q36.51) mean vs 99.99% (Q42.68)/99.98% (Q36.75) for PacBio CLR FALCON + Quiver + Pilon, 99.99% (Q44.95)/99.98% (Q37.28) for PacBio HiFi w/ Canu, 99.98% (Q38.12)/99.90% (Q30.30) for 10x Genomics w/ Supernova, and 99.77% (Q26.34)/99.72% (Q25.60) for GRCh38. Using the 4 BACs we associated to chrX, the identities are 99.99% (Q40.53) median/99.99% (Q40.89) mean vs 99.99% (Q42.60)/99.99% (Q42.04) for PacBio CLR FALCON + Quiver + Pilon, 99.99% (Q44.15)/99.99% (Q43.47) for PacBio HiFi w/ Canu, 99.99% (Q38.30)/99.98% (Q37.42) for 10x Genomics w/ Supernova, and 99.82% (Q27.51)/99.83% (Q27.61) for GRCh38.

Unique BACs were defined as those originating from regions at least 10 kb away from the nearest known segmental duplication. These accessions are: AC275297.1, AC275300.1, AC270133.1, AC270118.1, AC270136.1, AC275290.1, AC279018.1, AC270119.1, AC278482.1, AC275298.1, AC270134.1, AC279070.1, AC270238.1, AC270117.1, AC270132.1, AC270122.1, AC270137.1, AC270115.1, AC275304.1, AC270145.1, AC270121.1, AC278741.1, AC275291.1, AC275285.1, AC270135.1, AC270131.1, AC278929.1, AC275301.1, AC270146.1, AC275305.1, AC270120.1. X-associated BACs were identified based on mapping to the assembly, those accessions are: AC275293.1, AC270146.1, AC270120.1, AC275305.1 which are all a subset of the above unique BACs.

We also estimated assembly quality by measuring concordance of the consensus sequence with mapped 10x Genomics / Illumina data. Using the 10x mapping procedure described above, the bam file was filtered for mapping quality >20 using samtools v1.9 with the command `samtools view -hb -q20`. Variants were called using Freebayes v1.3.1 with the command `freebayes --skip-coverage 648`

`asm.bam -v asm.bayes.vcf -f asm.fasta`, excluding regions with excessive read coverage (12 x mean = 648). Calls genotyped as 0/1 (with support for the assembly allele) were filtered out and the total bases changed (added/deleted/substituted) *B* was summed. Total bases with at least 3-fold and less than 648-fold coverage, *T,* were also tabulated and the QV computed as , resulting in an average consensus quality estimate of 99.9896% (Q39.83). Note that these FreeBayes parameters are more aggressive and will call more variants than those used for polishing (e.g. the FreeBayes polishing only corrects indels), but this validation is still somewhat circular and we view the BAC validation as more reliable. Using the same criteria, measuring the QV on the X chromosome resulted in 99.9953% (Q43.31).

Supplementary Note 5. Structural variant analysis

To compare our CHM13 assembly to GRCh38 as a reference for calling structural variation, contigs from several human assemblies were aligned to each of the two references with MUMmer version 3.23[14] (l=100, c=500), and structural variants were called using Assemblytics[15]. The four assemblies shown in Extended Data Fig3 are: (1) the maternal haplotype of NA12878[12] (2) TrioCanu assemblies of the maternal haplotypes of the Puerto Rican son HG00733 and the Yoruba son NA19240[16] and (3) a haplotype-phased assembly of a Korean individual[17]. When aligned to GRCh38, the four assemblies yield the following numbers of insertions/deletions: NA12878: 6785/4265, HG00733: 7861/4667, NA19240: 7993/5886, and AK1: 8176/5781. Aligned to the CHM13 assembly, the four assemblies give the following number of insertions/deletions: NA12878: 4129/4345, HG00733: 5018/4898, NA19240: 5707/6578, and AK1: 5657/6113. This excess of insertion calls with respect to GRCh38 exists across a wide size range, and is absent in calls against CHM13.

In addition to insertions and deletions, inversions were called against both the GRCh38 reference and our CHM13 assembly with SVrefine v0.34 (https://github.com/nhansen/SVanalyzer) using MUMmer alignments to the same four assemblies as used to call large insertions and deletions. SVrefine predicts a total of 102 inversions against GRCh38 (NA12878:16, HG00733:35, NA19240:26, and AK1:25), and 41 inversions against the CHM13 assembly (NA12878:5, HG00733:14, NA19240:16, and AK1:6). After merging equivalent calls with SVmerge, we manually curated 63 calls and genotyped them in all four of the assemblies by inspecting alignments to the inverted region of the reference. Assemblies were classified as matching the GRCh38 reference allele (REF), matching the inverted alternate allele (INV), having no coverage across the region (NoCov), or exhibiting alignments that neither match the GRCh38 reference nor the inversion (Complex). Supplementary Table 5 lists all confirmed inversion calls, of which 19 are unique to GRCh38 (possible reference errors) and 1 is unique to CHM13 (possible assembly error).

Supplementary Note 6. Determination of copy number of repetitive regions using droplet digital PCR (ddPCR)

Genomic DNA was isolated using DNeasy Blood & Tissue Kit (Qiagen). DNA was quantified using Qubit Fluorometer with Qubit dsDNA HS Assay (Invitrogen). 20uL reaction were

performed with 1 ng of gDNA, except for DXZ1 which was run with 0.1 ng of gDNA. Primers and restriction enzymes are listed in the supplementaryl table. EvaGreen ddPCR reactions were performed using the manufacturer's protocol (Bio-Rad). Mastermixes were simultaneously prepared for HPRT1 and the gene of interest which were then incubated for 15 minutes to allow for restriction digest. Statistics were performed using the confidence interval calculated by the Quantasoft software and applying it to Taylor's expansion.

Chromosome region	Forward Primer (5'-3')	Reverse primer (5'-3')	Restriction enzyme
CT45	CATCAGCCATGGTGGAGTAT	TGCGGTGTTTCCCTGTT	HaeIII
CT47	GAGATCGGACCCGATGATTC	CCAGTAAATCTCCCACCC	AluI
DXZ1	TGATAGCGCAGCTTTGACAC	TTCCAACACAGTCCTCCA	HaeIII
DXZ4	CACTTCTACCACCACGAGTAA	GGGATGACATTCAACTGGGA	AluI
GAGE	GTAACGGAGGTCGTGGATTA	CGCACTGAGAATAAGGGAG	AluI
Reference Gene	Forward Primer (5'-3')	Reverse primer (5'-3')	Restriction enzyme
HPRT1	AAGGTGCTGGTCTCCTTTAC	GCACCAATGATTCTCTCCCT	AluI

Supplementary Note 7. Chromosome X centromere (DXZ1) array PFGE Southern analysis

Pulsed field gel electrophoresis

Alpha satellite array sizes were estimated by PFGE and Southern blotting using established methods[18,19]. High molecular weight DNA from 10^7–10^8 was embedded in 1% low melting point agarose plugs and digested with restriction enzymes that cut infrequently within alpha satellite DNA, releasing the DXZ1 array as one of a few large fragments. HMW DNA in one-half of an agarose plug was digested overnight with 20U of enzyme and run on 1% agarose gel. *Saccharomyces cerevisiae* and *Hansenula wingei* chromosomes embedded in agarose were used as size standards (Bio-Rad CHEF DNA Size Markers). Gels were run at 3 volts/cm for 50 hours at 14 °C in 1X TAE buffer, using switch times of 250 seconds (initial) – 900 seconds (final). Cell lines containing previously sized DXZ1 arrays were used as controls[18–20].

Southern blotting

After electrophoresis, gels were stained with ethidium bromide and imaged using a UV light source. Gels were rinsed briefly with distilled water, depurinated with 0.25 M HCl for 12 minutes at room temperature, then incubated twice for 15 minutes in denaturing buffer (1.5 M NaCl, 0.5 M NaOH). DNA was transferred to HyBond-N+ membrane (GE Healthcare/Amersham) for 48 hours in fresh denaturing buffer. Dried membranes were UV crosslinked (auto-crosslink setting on Stratagene Stratalinker) before proceeding to hybridization.

A 500 bp fragment (2 micrograms) spanning monomers 9–12 of DXZ1 was generated by PCR[21] and labeled overnight at 37°C with digoxygenin-11-dUTP using DIG High Prime (Sigma-Aldrich). Alternatively, a plasmid containing an entire DXZ1 HOR (2 kbp) was labeled by nick translation with digoxygenin-11-dUTP for 90 minutes at 14 °C. Labeling reactions were purified using either the High Pure PCR purification kit (Roche) or G-50 sephadex columns.

Membranes were pre-hybridized for 30–45 minutes in glass hybridization bottles containing 20 mL ExpressHyb buffer (Clontech) at 63 °C. Pre-hybridization buffer was replaced with 20 mL of fresh ExpressHyb containing 300–400 ng of labeled probe that had been denatured at 95 °C for 10 minutes. The probe was allowed to hybridize to the membrane at 63 °C overnight in a hybridization oven. Membranes were washed at 68 °C twice for 20 minutes in 2X SSC/0.1% sodium dodecyl sulfate (SDS), followed by a single high-stringency wash in 0.2X SSC/0.1% SDS for 15 minutes at 68 °C. Membranes were blocked in 1x Western blocking reagent (Roche) in maleic acid buffer (0.1 M maleic acid, 0.15 M NaCl, pH 7.5) for 45–60 minutes at room temperature, then incubated for 30 minutes in blocking buffer with anti-digoxygenin-alkaline phosphatase (Roche, 1:2000). Chemiluminescent detection was performed using 4–5 mL of CDP-Star ready-to-use reagent (Tropix). Membranes were imaged on a G:Box using GeneSys software (Syngene) for direct image analysis. Images were adjusted (leveled to curves) and labeled in Adobe Photoshop.

Supplementary Note 8. Chromosome X centromere (DXZ1) CRISPR-Cas9 duplex sequencing

DXZ1 CRISPR-Cas9 in vitro digestion

CRISPR-DS was performed as previously described[22] for a single sample (CHM13). Briefly, we designed the following guide RNA sequences to excise the DXZ1 centromeric satellite DNA: GAGGGCTTTGAGGCCTGTGGTGG and GTTCCTTCCTATACGACCGTAGG. 30nM of gRNAs were incubated with Cas9 nuclease at 25 °C for 10 min. We used a 0.5X ratio of AMPure beads to size select for the excised DNA fragments. Then the fragments were A-tailed and ligated to adapters including a 10 bp random double-stranded molecular tag (TwinStrand Biosciences) using the NEB kit as described[23]. The ligated DNA was amplified using KAPA Real-Time Amplification kit with fluorescent standards (KAPA Biosystems). Two xGen Lockdown Probes (IDT) specific to DXZ1 (4 nmole Ultramer DNA Oligo, shown below) were used to perform hybridization capture as previously reported with minor modifications[22]. The lockdown probes

were pooled in equimolar amounts and diluted to 0.75 pmol/μL in low TE (0.1 mM EDTA).

/5Biosg/GAAACGACTTTGTGAGGATGGCATTCAACTCATGGAGTTGAACAATCCTATTGATAGAGCAGATTGGAATCACTCTTTTTGTAGAATCTGCAAATGGAGATTTGGACTGCTTTGAGGCCT

/5Biosg/GAGGCCTGTGGTGGAAAAGGAAATATCTTCACATAAAAACTAGATAGAAACACTCTGAGAAAGTTCTTCATGATGAATGCATTTAACTCGCAGAGATGAACCTGCCTTTGAGAGTTCAGG

The CHM13 sample was quantified using the Qubit dsDNA HS Assay Kit, diluted, and pooled for sequencing. The library was sequenced on the MiSeq Illumina platform using a v3 600 cycle kit (Illumina), as specified by the manufacturer. Analysis was performed as previously described[23] using software available: https://github.com/risqueslab

Supplementary Tables

Name	T2T X	T2T WG
GenesFound	841	19618
GenesFoundPercent	99.64	99.68
TranscriptsFound	2994	83332
TranscriptsFoundPercent	99.87	99.82
FullmRNACoverage	2628	71684
FullmRNACoveragePercent	87.66	85.87
FullCDSCoverage	2788	77114
FullCDSCoveragePercent	93.00	92.37
TranscriptsWithFrameshift	19	334
TranscriptsWithFrameshiftPercent	0.63	0.40
TranscriptsWithOriginalIntrons	2771	77927
TranscriptsWithOriginalIntronsPercent	92.43	93.35
TranscriptsWithFullCDSCoverage	2788	77114
TranscriptsWithFullCDSCoveragePercent	93.00	92.37
TranscriptsWithFullCDSCoverageAndNoFrameshifts	2788	77101
TranscriptsWithFullCDSCoverageAndNoFrameshiftsPercent	93.00	92.36
TranscriptsWithFullCDSCoverageAndNoFrameshiftsAndOriginalIntrons	2711	76632
TranscriptsWithFullCDSCoverageAndNoFrameshiftsAndOriginalIntronsPercent	90.43	91.80
GenesWithFrameshift	9	170
GenesWithFrameshiftPercent	1.07	0.86
GenesWithOriginalIntrons	803	18490

GenesWithOriginalIntronsPercent	95.14	93.95
GenesWithFullCDSCoverage	794	18314
GenesWithFullCDSCoveragePercent	94.08	93.06
GenesWithFullCDSCoverageAndNoFrameshifts	796	18355
GenesWithFullCDSCoverageAndNoFrameshiftsPercent	94.31	93.27
GenesWithFullCDSCoverageAndNoFrameshiftsAndOriginalIntrons	788	18330
GenesWithFullCDSCoverageAndNoFrameshiftsAndOriginalIntronsPercent	93.36	93.14
MissingGenes	3	62
MissingGenesPercent	0.36	0.32

Supplementary Table 1. Genome annotation results from the Comparative Annotation Toolkit (CAT) for the CHM13 assembly presented here. Results are provided for both chromosome X and the whole genome.

GRCh38 coordinates	CHM13 coordinates	Genotypes against GRCh38				
		CHM13	NA12878	HG00733	NA19240	AK1
chr1:26639317-26648762*	Super-Scaffold_445:26610925-26601490	INV	NoCov	INV	INV	INV
chr1:197787659-197788856	Super-Scaffold_434:48224141-48226338	REF	INV	REF	REF	REF
chr2:95761618-96062896	Super-Scaffold_44:14412303-14713591	INV	NoCov	NoCov	NoCov	NoCov
chr2:138246675-138251774	Super-Scaffold_460_1:103993675-103998729	INV	INV	REF	INV	INV
chr2:240675032-240693858	Super-Scaffold_460_1:1500854-1519643	INV	NoCov	INV	Complex	REF
chr3:44699477-44700815*	Super-Scaffold_441:46259750-46261088	INV	INV	INV	INV	INV
chr3:187413745-187428816*	Super-Scaffold_39:10852970-10868040	INV	NoCov	INV	INV	INV
chr4:40233407-40235439	Super-Scaffold_65:9510902-9507870	REF	REF	REF	INV	REF
chr4:87926012-87937547*	Super-Scaffold_59:45039340-45050890	INV	INV	INV	INV	INV
chr5:64464922-64483091	Super-Scaffold_251:116263006-116281221	INV	INV	Complex	REF	INV

chr5:179633980-179658566	Super-Scaffold_251:1817928-1842505	INV	NoCov	Complex	Complex	INV
chr6:106720678-106723661*	Super-Scaffold_21:46529717-46526732	INV	INV	INV	INV	INV
chr6:130527041-130531150	Super-Scaffold_21:70353149-70358258	REF	REF	REF	INV	REF
chr7:40839777-40840871	Super-Scaffold_55:17319237-17317143	REF	Complex	INV	REF	INV
chr7:54218128-54324271	Super-Scaffold_55:3937158-3830002	REF	NoCov	INV	REF	NoCov
chr7:107418024-107423294*	Super-Scaffold_100011:30661482-30656212	INV	INV	INV	INV	INV
chr8:6296690-6300942*	Super-Scaffold_100058:6052335-6048081	INV	INV	INV	INV	INV
chr8:111062868-111063733**	Super-Scaffold_36:34169919-34170784	INV	REF	REF	REF	REF
chr9:30951219-30957624	Super-Scaffold_100031:8332775-8325371	REF	REF	INV	REF	REF
chr9:123976373-123993772*	Super-Scaffold_304:14421317-14438716	INV	INV	NoCov	INV	INV
chr10:37102417-37113835	Super-Scaffold_45:37221671-37234087	REF	REF	REF	INV	INV
chr10:91440437-91449180*	Super-Scaffold_58:42419651-42428395	INV	INV	INV	INV	INV
chr11:310165-319615*	Super-Scaffold_100240:48731517-48740966	INV	NoCov	INV	INV	INV
chr11:50113122-50365466*	Super-Scaffold_452:882772-630322	INV	NoCov	INV	NoCov	NoCov
chr11:62093043-62102999*	Super-Scaffold_100238:8082674-8072706	INV	INV	INV	INV	INV
chr12:12391920-12393808*	Super-Scaffold_453:22417328-22419231	INV	INV	INV	INV	INV
chr12:13391597-13398661	Super-Scaffold_453:21407093-21414157	INV	NoCov	INV	Complex	Complex
chr12:17768363-17861562*	Super-Scaffold_453:16945928-17039175	INV	NoCov	INV	INV	INV

chr12:86845556-86860052	NA	Complex	INV	INV	Complex	INV
chr14:34540659-34562456	NA	Complex	NoCov	INV	Complex	REF
chr14:60604530-60613248	Super-Scaffold_43:44988673-44998393	REF	REF	REF	INV	REF
chr14:105693569-105700380	Super-Scaffold_43:90149105-90142271	INV	NoCov	Complex	REF	Complex
chr16:1229159-1255289	Super-Scaffold_491:1272664-1246551	INV	INV	Complex	INV	INV
chr16:75204667-75224294	Super-Scaffold_100044:29485516-29506144	REF	NoCov	INV	INV	INV
chr16:85155064-85156196	NA	Complex	INV	Complex	REF	INV
chr17:5982122-5983821*	Super-Scaffold_100043:5878375-5876676	INV	INV	INV	INV	INV
chr17:30616129-30631350	Super-Scaffold_100023:52830253-52845472	INV	REF	NoCov	INV	INV
chr18:12141452-12150154	Super-Scaffold_100046:3540284-3548990	INV	NoCov	INV	Complex	Complex
chr19:38769331-38793390*	Super-Scaffold_466:20036489-20060551	INV	NoCov	INV	INV	NoCov
chr20:10807399-10808852	Super-Scaffold_117:16039792-16041245	INV	REF	REF	Complex	REF
chr21:26648364-26649565	Super-Scaffold_460_2:13522137-13524338	REF	REF	REF	INV	REF
chr21:40022870-40039188*	Super-Scaffold_460_2:26942570-26926228	INV	NoCov	INV	INV	INV
chrX:52040715-52213380*	chrX_v0.7:50981131-51782187	INV	NoCov	INV	NoCov	NoCov
chrX:52881815-52973995	NA	Complex	NoCov	INV	NoCov	NoCov
chrX:55453508-55519694	chrX_v0.7:54821023-54754843	INV	NoCov	NoCov	NoCov	NoCov
chrX:76141833-76153276	chrX_v0.7:74594454-74583012	INV	NoCov	INV	REF	NoCov
chrX:101597510-101616279	chrX_v0.7:100049613-100069384	REF	NoCov	INV	REF	NoCov

chrX:106266280-106300079	chrX_v0.7:104707672-104742488	REF	NoCov	INV	INV	NoCov
chrX:141574144-141601876	NA	Complex	Complex	INV	REF	NoCov
chrX:149652865-149750398*	chrX_v0.7:148027045-147929535	INV	NoCov	INV	NoCov	NoCov
chrX:153106017-153293927	chrX_v0.7:151572349-151384454	INV	NoCov	NoCov	NoCov	NoCov
chrX:154555883-154648555	chrX_v0.7:152895217-152802484	INV	Complex	Complex	NoCov	NoCov

Supplementary Table 2. Curated inversion calls versus the GRCh38 reference and the CHM13 assembly, along with their genotypes in other assemblies. Genotype key: REF=assembly aligns to the GRCh38 reference without structural variation, INV=assembly displays a clear inversion with respect to the GRCh38 reference, NoCov=assembly has no coverage that spans the inverted region, and Complex=alignments of assembly contigs across the inverted region display neither the reference nor the inverted allele. The 19 potential inversion errors identified in the GRCh38 reference are marked with an asterisk in the first column of this table, and the single potential error in the CHM13 assembly is marked with two asterisks.

Assembly Name	Sample	Assembler	Cov	Instrument / Chemistry	# Ctg	Size (Gbp)	NG50 (Mbp)
GCA_000983475.1	CHM13	Celera Assembler	70x	RSII/P5+P6	10,430	3.00	5.35
GCA_000983455.2	CHM13	Falcon	70x	RSII/P5+P6	4,961	2.94	9.85
GCA_001015385.3	CHM13	Celera Assembler	70x	RSII/P5+P6	12,091	3.07	11.95
GCA_000983465.1	CHM13	Celera Assembler	70x	RSII/P5+P6	15,538	3.06	12.48
GCA_001015355.1	CHM13	Celera Assembler	70x	RSII/P5+P6	11,138	3.03	19.03
GCA_001307015.1	CHM1	Celera Assembler	120x	RSII/P5+P6	5,307	3.01	25.37
GCA_001297185.2	CHM1	Falcon	60x	RSII P6	3,709	3.00	26.13
GCA_001524155.4	NA19240	Falcon + BioNano	73x	RSII P6	2,439	2.87	28.15
GCA_002884485.1	CHM13	Falcon	76x	RSII P6	1,916	2.88	28.20
GCA_002180035.3	HG00514	Falcon + BioNano	80x	RSII P6	2,799	2.86	29.00
GCA_001420755.1	CHM1	Celera Assembler	120x	RSII/P5+P6	2,416	2.95	29.05
GCA_001420765.1	CHM1	Celera Assembler	120x	RSII/P5+P6	3,188	2.99	32.45

GCA_000001405.28	GRCh38p13	N/A	N/A	N/A	1,590	3.11	56.41
T2T v0.6	CHM13	Canu	39x + 70x	Oxford GridION/9.4.1	590	2.93	71.7

Supplementary Table 3. All human genome assemblies in NCBI with contig NG50 >25 Mbp or originating from CHM13. Sequences were downloaded from the FTP site and scaffolds split at 3 consecutive Ns to get contigs. Ns were excluded from the genome size of each assembly. A genome size of 3.0988 was used for computing NG50 for all assemblies. Aside from the Nanopore assembly presented here, all other assemblies in the table were generated using PacBio CLR data. The CHM13 PacBio CLR assembly we compare against in the main text is GCA_002884485.1 which had the highest score for BAC resolution of all CHM13 assemblies tested and incorporated the highest coverage PacBio data.

Cell line	PFGE DXZ1 Estimation	ddPCR DXZ1 Estimation
HAP1	3.7 Mb	3.7 Mb
t60-12	3.0-3.1 Mb	3.2 Mb
HDF	3.8 Mb	2.9 Mb
LT690	1.5 Mb	1.4 Mb
CHM13	2.8 Mb	2.8 Mb

Supplementary Table 4. DXZ1 array estimations for five different cell lines using PFGE and ddPCR. *HPRT1* was used as ddPCR single copy reference gene. PFGE were the result of at least three different runs with several standards.

Sequence Name	RefStartPos	RefEndPos	Type	Size
chrX_bothkpatchedin	48,733,807	48,790,958	insertion	124,036
chrX_bothkpatchedin	70,270,806	70,340,885	deletion	25,207
chrX_bothkpatchedin	106,136,920	106,142,580	insertion	2,961
chrX_bothkpatchedin	133,151,139	133,220,191	insertion	17,489

Supplementary Table 5. Structural variants identified by BioNano optical map in chromosome X draft. A table displaying coordinates and sizes of SVs identified in the candidate chromosome

X draft.

References

1. Alexander, D. H., Novembre, J. & Lange, K. Fast model-based estimation of ancestry in unrelated individuals. *Genome Res.* **19**, 1655–1664 (2009).
2. 1000 Genomes Project Consortium *et al.* A global reference for human genetic variation. *Nature* **526**, 68–74 (2015).
3. Mallick, S. *et al.* The Simons Genome Diversity Project: 300 genomes from 142 diverse populations. *Nature* **538**, 201–206 (2016).
4. Li, H. A statistical framework for SNP calling, mutation discovery, association mapping and population genetical parameter estimation from sequencing data. *Bioinformatics* **27**, 2987–2993 (2011).
5. Chang, C. C. *et al.* Second-generation PLINK: rising to the challenge of larger and richer datasets. *Gigascience* **4**, 7 (2015).
6. Jain, M. *et al.* Nanopore sequencing and assembly of a human genome with ultra-long reads. doi:10.1101/128835.
7. Chin, C.-S. *et al.* Nonhybrid, finished microbial genome assemblies from long-read SMRT sequencing data. *Nat. Methods* **10**, 563–569 (2013).
8. Kronenberg, Z. N. *et al.* High-resolution comparative analysis of great ape genomes. *Science* **360**, (2018).
9. Koren, S. *et al.* Canu: scalable and accurate long-read assembly via adaptive k-mer weighting and repeat separation. *Genome Res.* **27**, 722–736 (2017).
10. Kolmogorov, M., Yuan, J., Lin, Y. & Pevzner, P. A. Assembly of long, error-prone reads using repeat graphs. *Nat. Biotechnol.* **37**, 540–546 (2019).

11. Vollger, M. R. *et al.* Long-read sequence and assembly of segmental duplications. *Nat. Methods* **16**, 88–94 (2019).

12. Koren, S. *et al.* De novo assembly of haplotype-resolved genomes with trio binning. *Nat. Biotechnol.* (2018) doi:10.1038/nbt.4277.

13. Sedlazeck, F. J. *et al.* Accurate detection of complex structural variations using single-molecule sequencing. *Nat. Methods* **15**, 461–468 (2018).

14. Kurtz, S. *et al.* Versatile and open software for comparing large genomes. *Genome Biol.* **5**, R12 (2004).

15. Nattestad, M. & Schatz, M. C. Assemblytics: a web analytics tool for the detection of variants from an assembly. *Bioinformatics* **32**, 3021–3023 (2016).

16. Chaisson, M. J. P. *et al.* Multi-platform discovery of haplotype-resolved structural variation in human genomes. *Nat. Commun.* **10**, 1784 (2019).

17. Seo, J.-S. *et al.* De novo assembly and phasing of a Korean human genome. *Nature* **538**, 243–247 (2016).

18. Sullivan, L. L., Boivin, C. D., Mravinac, B., Song, I. Y. & Sullivan, B. A. Genomic size of CENP-A domain is proportional to total alpha satellite array size at human centromeres and expands in cancer cells. *Chromosome Res.* **19**, 457–470 (2011).

19. Mahtani, M. M. & Willard, H. F. Pulsed-field gel analysis of alpha-satellite DNA at the human X chromosome centromere: high-frequency polymorphisms and array size estimate. *Genomics* **7**, 607–613 (1990).

20. Mahtani, M. M. & Willard, H. F. Physical and genetic mapping of the human X chromosome centromere: repression of recombination. *Genome Res.* **8**, 100–110 (1998).

21. Mravinac, B. *et al.* Histone modifications within the human X centromere region. *PLoS One* **4**, e6602 (2009).

22. Nachmanson, D. *et al.* CRISPR-DS: an efficient, low DNA input method for ultra-accurate sequencing. *bioRxiv* 207027 (2017) doi:10.1101/207027.

23. Nachmanson, D. *et al.* Targeted genome fragmentation with CRISPR/Cas9 enables fast and efficient enrichment of small genomic regions and ultra-accurate sequencing with low DNA input (CRISPR-DS). *Genome Research* vol. 28 1589–1599 (2018).
