# Supplementary material for: Telomere-to-telomere assembly of a complete human X chromosome
Source: Nature. 2020 Jul 14;585(7823):79–84. doi: 10.1038/s41586-020-2547-7 (PMC7484160; doi:10.1038/s41586-020-2547-7)
Supplement: Supplementary file 2 — Reporting Summary [file 41586_2020_2547_MOESM2_ESM.pdf]

nature research

Corresponding author(s): Karen H. Miga and Adam M. Phillippy

Last updated by author(s): Feb 2, 2020

Reporting Summary

Nature Research wishes to improve the reproducibility of the work that we publish. This form provides structure for consistency and transparency in reporting. For further information on Nature Research policies, see Authors & Referees and the Editorial Policy Checklist .

Statistics

For all statistical analyses, confirm that the following items are present in the figure legend, table legend, main text, or Methods section.

n/a	Confirmed	
☐	☒	The exact sample size (*n*) for each experimental group/condition, given as a discrete number and unit of measurement
☐	☒	A statement on whether measurements were taken from distinct samples or whether the same sample was measured repeatedly
☒	☐	The statistical test(s) used AND whether they are one- or two-sided<br>*Only common tests should be described solely by name; describe more complex techniques in the Methods section.*
☒	☐	A description of all covariates tested
☒	☐	A description of any assumptions or corrections, such as tests of normality and adjustment for multiple comparisons
☒	☐	A full description of the statistical parameters including central tendency (e.g. means) or other basic estimates (e.g. regression coefficient) AND variation (e.g. standard deviation) or associated estimates of uncertainty (e.g. confidence intervals)
☒	☐	For null hypothesis testing, the test statistic (e.g. *F*, *t*, *r*) with confidence intervals, effect sizes, degrees of freedom and *P* value noted<br>*Give P values as exact values whenever suitable.*
☒	☐	For Bayesian analysis, information on the choice of priors and Markov chain Monte Carlo settings
☒	☐	For hierarchical and complex designs, identification of the appropriate level for tests and full reporting of outcomes
☒	☐	Estimates of effect sizes (e.g. Cohen's *d*, Pearson's *r*), indicating how they were calculated

Our web collection on statistics for biologists contains articles on many of the points above.

Software and code

Policy information about availability of computer code

Data collection	MinKNOW (version 3.4.5) software, base calling was performed using Guppy (flip-flop version 2.3.1), 10XG raw data was processed using RTA3.3.3,
Data analysis	Canu 1.7.1, bwa0.7.12, Minimap2 v2.71-941, Arrow v2.2.2 from SMRTlink 6.0.0.47841, Nanopolish v0.11.0, hmmer v3, Supernova v2.1.1, Long Ranger v2.2.2 , Juicer v1.5.6, maps were visualized with Juicebox v1.8.8, Meryl from Canu v1.8, Flye 2.4, samtools v1.9, freebayes v1.2.0 and v1.3.1, MUMmer version 3.23, available CRISPR-DS software (https://github.com/risqueslab)

For manuscripts utilizing custom algorithms or software that are central to the research but not yet described in published literature, software must be made available to editors/reviewers. We strongly encourage code deposition in a community repository (e.g. GitHub). See the Nature Research guidelines for submitting code & software for further information.

Data

Policy information about availability of data

All manuscripts must include a data availability statement. This statement should provide the following information, where applicable:

- Accession codes, unique identifiers, or web links for publicly available datasets
- A list of figures that have associated raw data
- A description of any restrictions on data availability

Original data generated at SIMR that underlies this manuscript can be accessed from the Stowers Original Data Repository at http://www.stowers.org/research/publications/libpb-1453. Genome assemblies and sequencing data including raw signal files (FAST5), event-level data (FAST5), base-calls (FASTQ), and alignments (BAM/CRAM) are available as an Amazon Web Services Open Data set. Instructions for accessing the data, as well as future updates to the raw data and assembly, are available from https://github.com/nanopore-wgs-consortium/chm13. All data is additionally archived and available under NCBI BioProject accession PRJNA559484 including the whole-genome assembly (GCA_009914755.1) and completed X chromosome (CM020874.1).

Field-specific reporting

Please select the one below that is the best fit for your research. If you are not sure, read the appropriate sections before making your selection.

☒ Life sciences ☐ Behavioural & social sciences ☐ Ecological, evolutionary & environmental sciences

For a reference copy of the document with all sections, see nature.com/documents/nr-reporting-summary-flat.pdf

Life sciences study design

All studies must disclose on these points even when the disclosure is negative.

Sample size	Only one cell line (CHM13) is used in this study to reduce the complexity of repeat assembly
Data exclusions	No data was excluded from this study
Replication	ddPCR copy number estimates were performed in triplicate, cytogenetic assessement were performed over ten metaphase spreads, pulsed-field gel Southern experiments were performed with technical replicates
Randomization	This is not relevant to our work, no randomization was performed as we are using one sample
Blinding	Blinding was not relevant to this work

Reporting for specific materials, systems and methods

We require information from authors about some types of materials, experimental systems and methods used in many studies. Here, indicate whether each material, system or method listed is relevant to your study. If you are not sure if a list item applies to your research, read the appropriate section before selecting a response.

Materials & experimental systems

n/a	Involved in the study	
☒	☐	Antibodies
☐	☒	Eukaryotic cell lines
☒	☐	Palaeontology
☒	☐	Animals and other organisms
☒	☐	Human research participants
☒	☐	Clinical data

Methods

n/a	Involved in the study	
☒	☐	ChIP-seq
☒	☐	Flow cytometry
☒	☐	MRI-based neuroimaging

Eukaryotic cell lines

Policy information about cell lines

Cell line source(s)	Cells from a case of a complete hydatidiform mole CHM13 were cultured, karyotyped using Q banding and cryopreserved at Magee-Womens Hospital (Pittsburgh, PA).
Authentication	The CHM13 line was authenticated by cytogenetic analysis (G-banding and SKY) before use. No contamination was identified.
Mycoplasma contamination	CHM13 has been determined to be negative for Mycoplasma contamination
Commonly misidentified lines (See ICLAC register)	N/A
